# Optimizing Indocyanine Green Fluorescence Cholangiography in Laparoscopic Cholecystectomy: A Systematic Review of Randomized Controlled Trials on Dose, Timing, Route, Efficacy, and Safety

**DOI:** 10.3390/medicina62102022

**Published:** 2026-10-19

**Authors:** Dimitrios Tsavdaris, Georgios Geropoulos, Anastasios Gialouris, Alexandros Mekras, Kyriakos Psarras, Daniel Paramythiotis

**Affiliations:** 1 First Department of Propaedeutic Surgery, University General Hospital of Thessaloniki AHEPA, Aristotle University of Thessaloniki, 54636 Thessaloniki, Greecetasosgialouris1999@gmail.com (A.G.); 2 Third Department of Surgery, University General Hospital of Thessaloniki AHEPA, Aristotle University of Thessaloniki, 54636 Thessaloniki, Greecepsarrask@auth.gr (K.P.); 3 Department of General and Visceral Surgery, SHG-Klinikum Merzig, Academic Hospital of University of Saarland, 66663 Merzig, Germany

**Keywords:** indocyanine green, fluorescence cholangiography, laparoscopic cholecystectomy, near-infrared imaging, biliary anatomy, systematic review

## Abstract

*Background and Objectives*: Near-infrared fluorescence cholangiography with indocyanine green (ICG) has been proposed as an adjunct for improving biliary anatomy recognition during laparoscopic cholecystectomy. However, uncertainty remains regarding its comparative efficacy, operative value, safety, and optimal route, dose, and timing. *Materials and Methods*: A systematic review was conducted to identify randomized comparative and dose-finding studies evaluating ICG fluorescence cholangiography during laparoscopic cholecystectomy. Outcomes included visualization of the cystic duct, common hepatic duct, common bile duct, biliary junctions, operative time, time to critical anatomical identification, conversion or bailout procedures, length of stay, bile duct injury, bile leak, and ICG-related adverse events. Clinical and methodological heterogeneity precluded meta-analysis; findings were synthesized descriptively. *Results*: Eighteen studies were included. ICG improved visualization of selected biliary structures compared with white-light imaging, particularly before dissection, whereas differences versus radiographic intraoperative cholangiography were less pronounced. Operative effects were inconsistent: several studies reported shorter cholangiography duration or earlier anatomical identification, without consistent reductions in total operative time or length of stay. Bile duct injuries and bile leaks were infrequent, but event numbers were insufficient to establish a protective effect or comparative safety. Selected dose comparisons suggested reduced hepatic background fluorescence with lower doses, although findings depended on administration timing and imaging platform. Interpretation was limited by subjective visualization assessment, risk of bias, and heterogeneous patient populations. *Conclusions*: ICG fluorescence cholangiography improves visualization of selected biliary structures, but improved visualization should not be equated with demonstrated clinical benefit. Evidence remains insufficient to establish reductions in bile duct injury, conversion, or postoperative morbidity, or a universally optimal route, dose, or timing regimen.

## 1. Introduction

Laparoscopic cholecystectomy (LC) has become the treatment of choice for gallbladder removal, reserving open cholecystectomy (OC) for technically demanding cases, severe inflammatory disease, or conversion because of intraoperative difficulty or complications. LC offers many advantage over OC, including reduced postoperative pain, shorter hospital stay, faster functional recovery, and lower overall morbidity and mortality [1,2]. Despite these advantages, safe identification of biliary anatomy remains a critical determinant of operative success, particularly in cases with inflammation, adhesions, obesity, or anatomical variation. Consequently, several adjunctive imaging modalities have been introduced to improve intraoperative orientation and reduce the risk of bile duct injury, among which indocyanine green (ICG) fluorescence cholangiography has gained increasing attention.

ICG is a water-soluble fluorescent dye that in recent years seems to be used more as a method of marking various anatomical structures [3]. Specifically, when indocyanine is administered and under near-infrared light, ICG emits fluorescence, allowing real-time visualization of bile-containing structures without the need for duct cannulation or ionizing radiation [4]. Thus, by using indocyanine, we can now distinguish tissues and structures with greater precision, especially in cases where the anatomy of the area is varied or altered. In LC, ICG helps the surgeon to distinguish the anatomy of the bile ducts, a valuable help, given the great heterogeneity that the anatomy of the biliary system presents among patients, thus supporting a safer dissection, enhancing achievement of the critical view of safety (CVS), reducing reliance on intraoperative cholangiography in selected cases, and ultimately decreasing the risk of iatrogenic biliary injury [5,6].

However, although the use of ICG fluorescence cholangiography in LC has expanded considerably, several clinically relevant questions remain unresolved. Existing studies vary substantially with respect to ICG dose, timing of administration, route of delivery, imaging systems, outcome definitions, and patient populations. In particular, the optimal balance between adequate biliary fluorescence and excessive hepatic background signal remains uncertain, and the comparative effectiveness of different administration protocols has not been definitively established. Furthermore, while ICG is generally considered safe, a rigorous synthesis of safety outcomes remains important, especially as its use becomes more widespread. Therefore, this systematic review aims to evaluate the efficacy and safety of ICG fluorescence cholangiography in LC, with particular emphasis on the influence of dose, timing, and route of administration on biliary visualization and operative outcomes. By synthesizing evidence from randomized controlled trials, this study seeks to clarify the clinical value of ICG fluorescence cholangiography and identify protocol-related factors that may optimize its application in contemporary laparoscopic biliary surgery.

## 2. Materials and Methods

### 2.1. Study Design and Reporting

This systematic review was conducted in accordance with PRISMA recommendations [7,8] (Supplementary Table S1) and the Cochrane Handbook for Systematic Reviews of Interventions, and the protocol was registered in advance (https://doi.org/10.17605/OSF.IO/Z75DB). The review evaluated randomized controlled trials investigating indocyanine green (ICG)-based fluorescence imaging during laparoscopic cholecystectomy, including comparisons of ICG fluorescence cholangiography versus conventional techniques, intravenous versus intracholecystic ICG administration, and different ICG dose or timing strategies. Although this review was initially designed as a systematic review with meta-analysis, formal quantitative synthesis was not pursued because the included studies showed substantial clinical, methodological, and outcome-level heterogeneity. The studies differed in route of ICG administration, dose, timing of injection, comparator group, operative setting, imaging platform, and outcome definitions. In addition, biliary visualization was inconsistently reported across anatomical structures and procedural phases, with several studies reporting scores, ratios, qualitative grades, time-to-visualization outcomes, or incomplete duct-specific event data rather than a common endpoint. Given these differences, statistical pooling would have required substantial assumptions and would likely have generated summary estimates with limited clinical interpretability. We therefore prioritized a structured descriptive synthesis supported by study-level tables and evidence maps, allowing preservation of the direction, magnitude, and context of the reported findings without imposing inappropriate quantitative aggregation. Formal assessment of publication bias was also not undertaken. The heterogeneous intervention comparisons and outcome measures did not support a meaningful review-wide analysis of comparable effect estimates and their standard errors. Combining all included trials in a single funnel plot would therefore have been difficult to interpret.

### 2.2. Eligibility Criteria

Eligible studies were randomized controlled trials enrolling adult patients undergoing laparoscopic cholecystectomy in whom ICG fluorescence imaging was used for intraoperative visualization of biliary anatomy. Studies were included if they reported at least one relevant intraoperative visualization, operative, or safety outcome. Eligible comparators included white-light imaging, conventional laparoscopic cholecystectomy, intraoperative cholangiography, X-ray cholangiography, alternative ICG routes, or alternative ICG dosing/timing regimens. Non-randomized studies, observational cohorts, case series, reviews, editorials, conference-only abstracts without extractable data, and studies without relevant outcome data were excluded.

### 2.3. Outcomes

The primary efficacy outcomes were visualization of the cystic duct (CD), common hepatic duct (CHD), common bile duct (CBD), and biliary confluence/junction. Visualization outcomes were analyzed as binary events when reported as visible/not visible and as continuous outcomes when reported using visualization scores or fluorescence intensity metrics. Secondary outcomes included total operative time, cholangiography or imaging time, time to CVS, length of stay, conversion or bailout procedures, overall complications, bile duct injury, bile leak, biliary complications, puncture-site or route-specific events, and ICG-related adverse events. Dose-optimization outcomes included duct visualization scores, adequate bile duct visualization, bile-duct-to-liver fluorescence ratio, liver fluorescence intensity, and high-quality imaging outcomes.

### 2.4. Information Sources, Search Strategy, Selection Process and Data Extraction

The search was conducted in three databases, MEDLINE, Scopus and CENTRAL, from inception until 6 October 2026, employing the search terms analyzed in Supplementary Table S2. No search filter was used and there was no time limit. The selection process involved two independent reviewers (D.T. and D.P.) who assessed the eligibility of studies for inclusion in this systematic review and meta-analysis based on the title, abstract, and full-text evaluation.

For each eligible trial, two reviewers extracted study-level and arm-level data, including author, year, sample size, ICG dose, route of administration, timing of administration, comparator, patient age, sex distribution, body mass index, preoperative diagnosis, and reported outcomes. For binary outcomes, the number of events and total participants per arm were extracted. For continuous outcomes, means, standard deviations, and sample sizes were extracted. For dose–response analyses, dose was harmonized in milligrams where possible, and dose categories were defined as microdose, very low, low, intermediate, standard, and high dose according to the administered ICG amount and the distribution of available trial arms.

### 2.5. Risk-of-Bias Assessment and Certainty-of-Evidence Assessment

Risk of bias was assessed using the Cochrane RoB 2 tool for randomized trials. The following domains were evaluated: bias arising from the randomization process, bias due to deviations from intended interventions, bias due to missing outcome data, bias in outcome measurement, and bias in selection of the reported result. Each study was rated as low risk, some concerns, or high risk of bias overall.

Certainty of evidence was assessed using the Grading of Recommendations Assessment, Development and Evaluation (GRADE) approach, following the GRADE handbook. Assessments were performed separately for each outcome and intervention comparison rather than for individual studies. Randomized evidence initially received a high-certainty rating and was downgraded for serious or very serious concerns regarding risk of bias, inconsistency, indirectness, imprecision, or publication bias. Because meta-analysis was not performed, judgments were informed by the narrative synthesis, study-level effect estimates and reported confidence intervals, sample sizes, event counts, and differences in populations, interventions, and outcome definitions. Visualization outcomes were assessed separately from patient-important clinical outcomes, and dose–timing comparisons were interpreted within the tested regimens and imaging systems. Publication bias was considered qualitatively; formal asymmetry tests were not undertaken because comparable outcome-specific evidence was limited. Certainty was classified as high, moderate, low, or very low, with reasons for downgrading documented in the evidence profile [9].

## 3. Results

### 3.1. Study Selection

**Figure 1:**
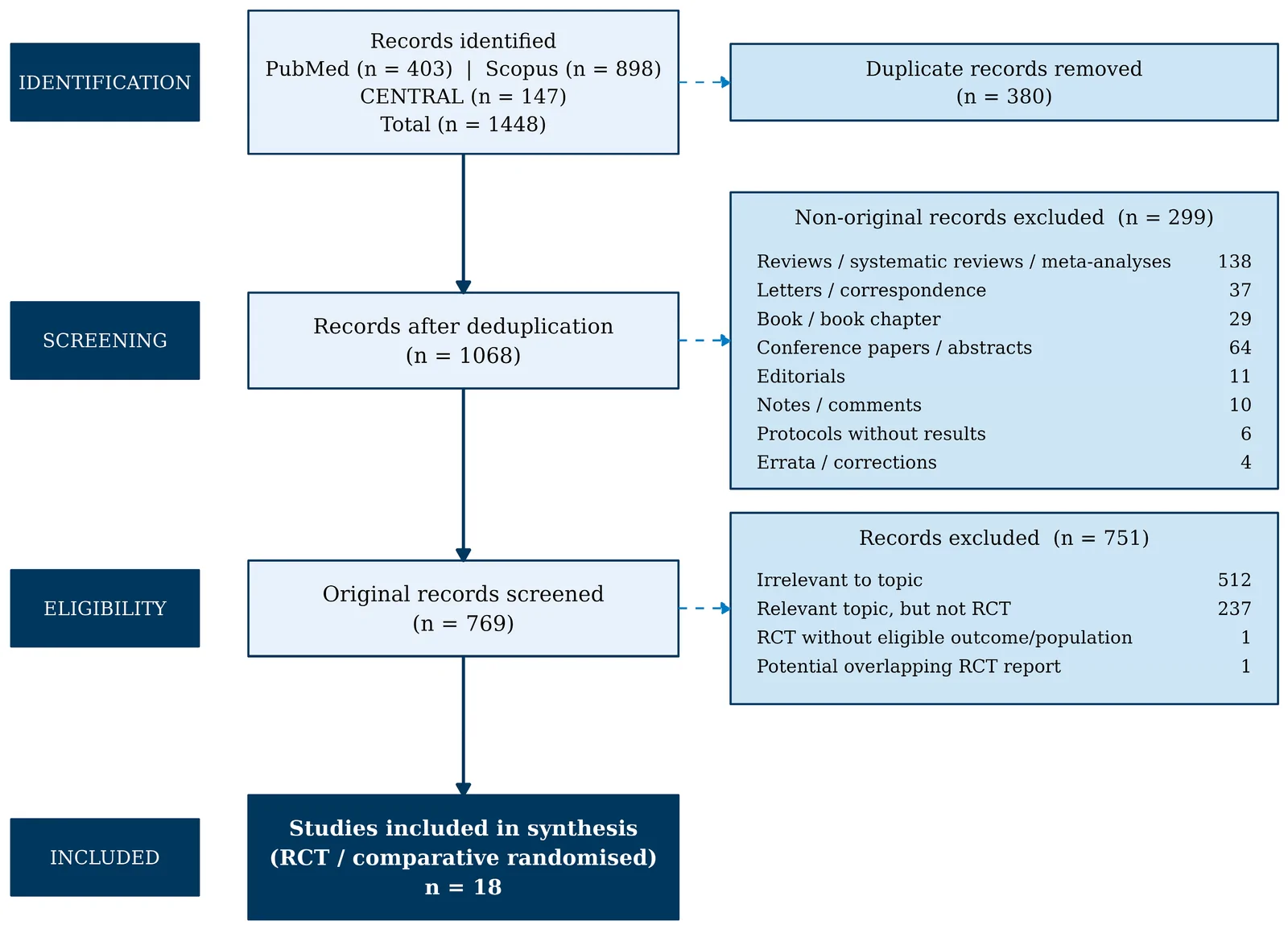
Prisma flowchart.

The database search identified 1448 records: 403 from PubMed, 898 from Scopus and 147 from CENTRAL (Figure 1). After removal of 380 duplicates, 1068 unique records were screened. Of these, 299 were excluded as non-original publications, including reviews, letters, editorials, comments, conference papers, protocols without results, book chapters, and errata. Among the remaining 769 original records, 512 were excluded as irrelevant to the review question and 237 were relevant to the broader topic but were not randomized trials. One randomized trial was excluded because it did not assess the target population or outcomes of interest, and one additional record was considered a potential overlapping report of an included trial. Overall, 18 studies met the eligibility criteria and were included in the qualitative synthesis. No formal meta-analysis was performed because of substantial heterogeneity in ICG protocol, comparator, imaging platform, surgical population, and outcome definitions. The study selection process is shown in the PRISMA flow diagram.

### 3.2. Study Characteristics

Across the 18 included randomized studies [10,11,12,13,14,15,16,17,18,19,20,21,22,23,24,25,26,27], the evidence base comprised 2832 patients undergoing laparoscopic cholecystectomy across elective, urgent/emergency, mixed-indication, route-comparison, timing-optimization, and dose-optimization settings (Table 1). Most trials enrolled patients undergoing LC for benign gallbladder disease, predominantly cholelithiasis, biliary colic, chronic cholecystitis, acute cholecystitis, gallbladder polyps, choledocholithiasis, gallstone pancreatitis, or related biliary conditions.

The studies could be grouped into four major methodological categories. First, several trials compared ICG fluorescence cholangiography with conventional techniques, including standard intraoperative cholangiography, X-ray cholangiography, white-light imaging, or conventional laparoscopic cholecystectomy. Second, three studies directly compared intravenous versus intracholecystic ICG administration [10,26,27]. Third, several trials focused on dose optimization, using fixed-dose, microdose, or BMI-adjusted dosing strategies [16,17,19,20,21]. Fourth, timing-focused studies evaluated administration intervals ranging from intraoperative injection to 24 h before surgery [11,22].

Baseline demographic characteristics were generally well balanced within individual trials. Mean or median age ranged from young adult cohorts in the route-comparison studies to older populations in acute cholecystitis and dose-optimization studies. Female predominance was common, although several trials had nearly balanced sex distributions. BMI was reported in most studies and generally ranged from the mid-20s to low-30s kg/m^2^, with higher BMI populations in selected dose and emergency cohorts.

ICG administration varied substantially across the included studies. Conventional-comparator trials used ICG fluorescence cholangiography against IOC, X-ray cholangiography, WLI, or conventional LC. Route-comparison trials evaluated intravenous versus intracholecystic administration. Optimization trials evaluated ICG timing from intraoperative injection to 24 h preoperatively, as well as fixed, microdose, standard-dose, and BMI-adjusted dosing strategies.

### 3.3. Visualization of Biliary Anatomy

**Figure 2:**
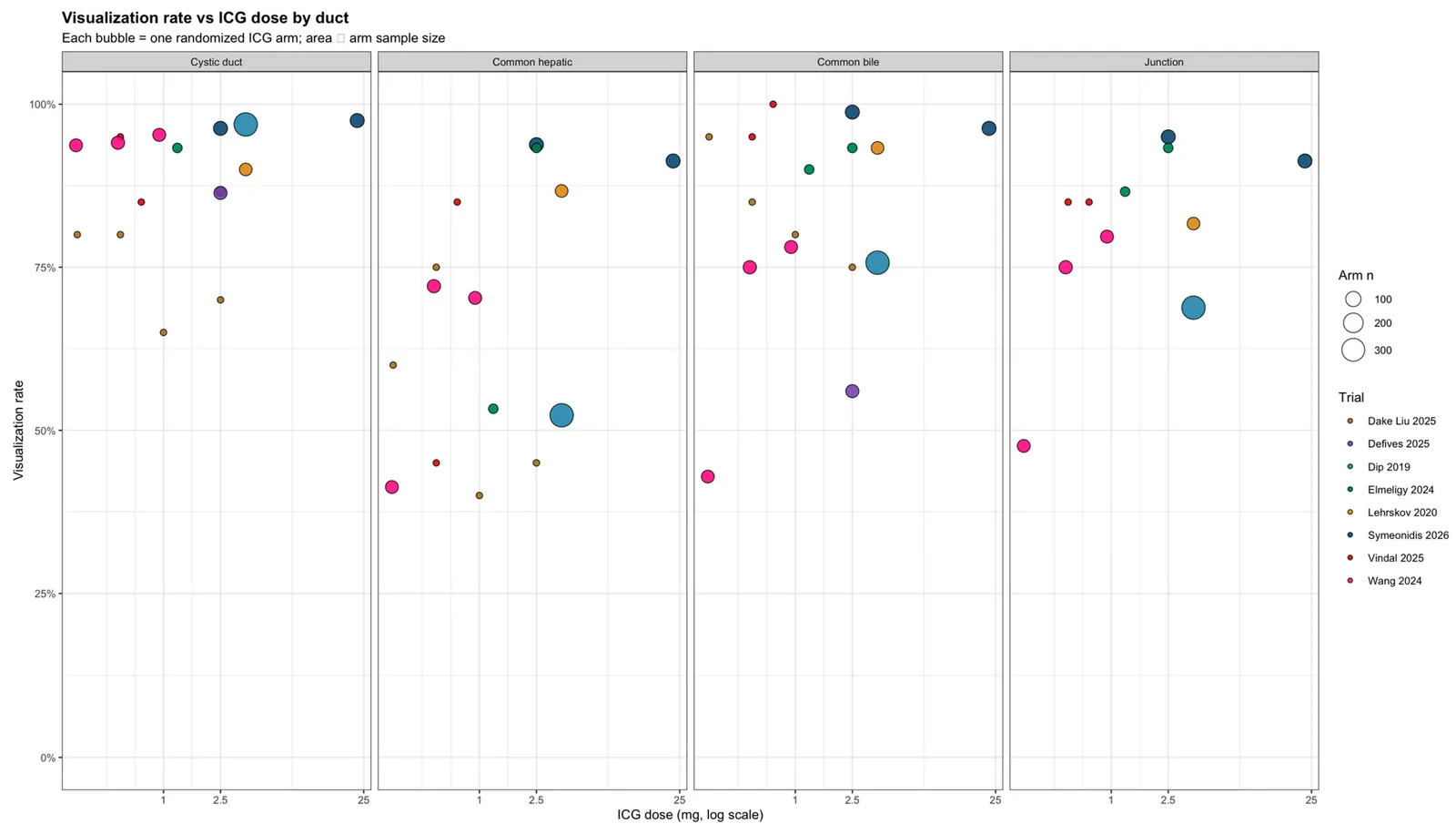
Visualization rate versus ICG dose by duct. Bubble plot of raw arm-level visualization rates for the cystic duct, common hepatic duct, common bile duct, and biliary junction/confluence. Each bubble represents one ICG arm; bubble area is proportional to arm sample size. The *x*-axis shows ICG dose on a log scale. Colors identify individual trials. No pooled estimates are shown. ICG (indocyanine green) [10,12,13,20,23,24,26,27].

ICG fluorescence cholangiography provided high visualization rates for the main extrahepatic biliary structures across most included studies, although performance varied by anatomical target and comparator (Table 2). Cystic duct visualization was generally the most consistent endpoint, frequently exceeding 85–95% in ICG arms. In Symeonidis 2026 [10], cystic duct visualization was 97.5% with IV ICG and 96.3% with intracholecystic ICG, compared with 93.5% with IOC. Similarly high rates were reported by Lehrskov 2020 [13] for IV ICG versus X-ray IOC, with cystic duct visualization of 90% versus 85%, respectively (Table 2; Figure 2).

**Figure 3:**
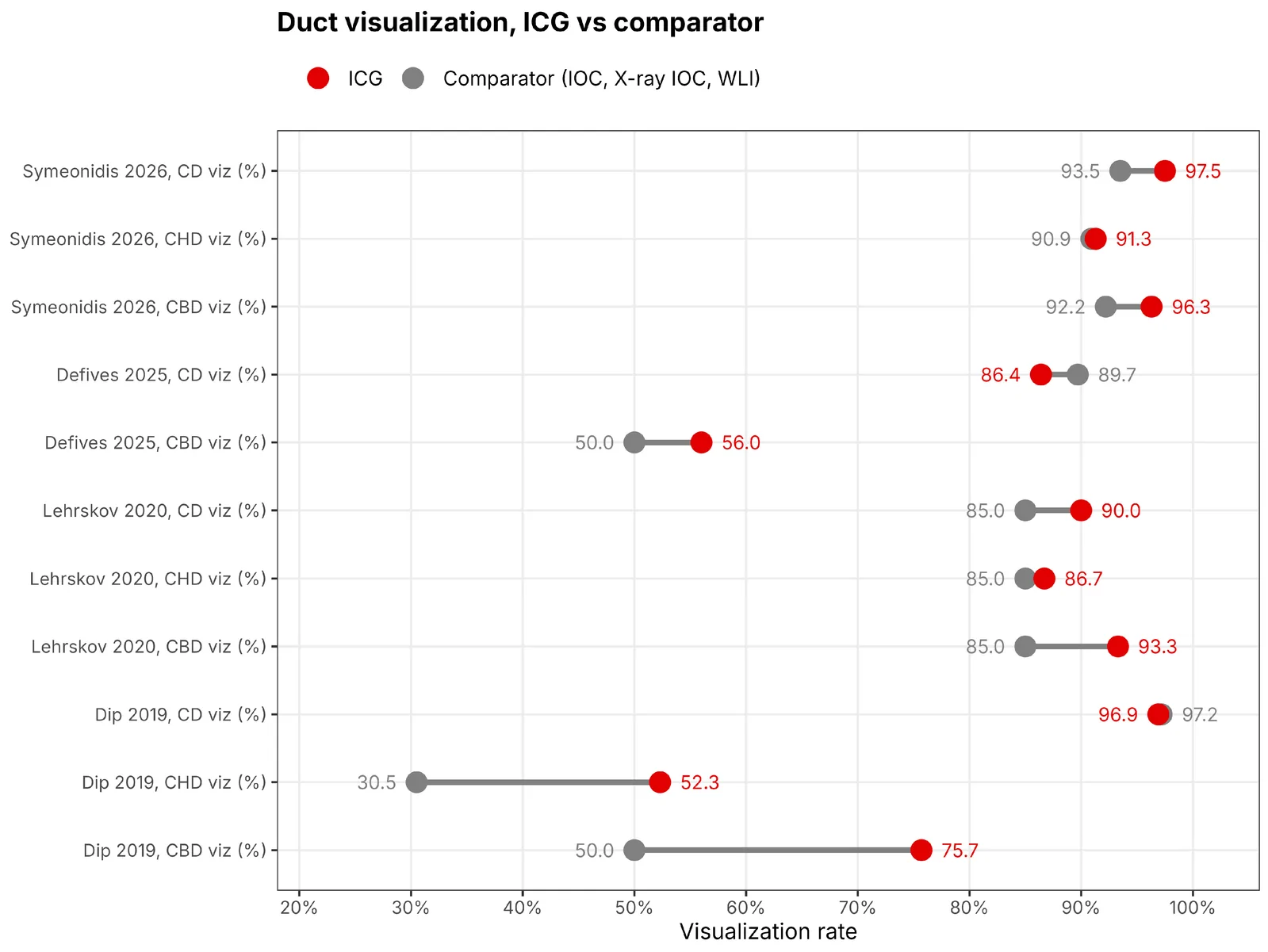
Duct visualization: ICG versus comparator [10,12,13,24]. Dumbbell plot of paired visualization rates for ICG versus comparator groups. Red points represent ICG; gray points represent comparator. Lines connect paired observations within the same study and duct-specific outcome.

Common bile duct visualization also tended to favor ICG in several datasets. Symeonidis 2026 [10] reported CBD visualization of 96.3% with IV ICG and 98.8% with intracholecystic ICG versus 92.2% with IOC, while Lehrskov 2020 [13] reported 93.3% with ICG versus 85.0% with X-ray IOC. The largest difference was observed in Dip 2019 [12], where post-dissection CBD visualization was 75.7% with near-infrared fluorescence cholangiography versus 50.0% with white-light imaging (Table 2; Figure 3). In the paired visualization plot, most CBD comparisons shifted in favor of ICG, indicating that the benefit was most apparent when fluorescence was compared with white-light visualization rather than with IOC (Supplementary Figure S1).

Common hepatic duct visualization was more variable. In Symeonidis 2026 [10], CHD visualization was high and similar across groups: 91.3% with IV ICG, 93.8% with intracholecystic ICG, and 90.9% with IOC. Lehrskov 2020 [13] likewise showed comparable CHD visualization with ICG and X-ray IOC, 86.7% versus 85.0%. By contrast, Dip 2019 [12] showed a clearer advantage for fluorescence over white-light imaging, with post-dissection CHD visualization of 52.3% versus 30.5% (Table 2; Figure 3). These findings suggest that ICG is most useful for CHD identification when the comparator is conventional white-light imaging, whereas its advantage over IOC is less pronounced.

Junctional anatomy was less consistently reported, but available data support a similar pattern. Symeonidis 2026 [10] showed high critical junction visualization across all groups, ranging from 91.3% to 95.0%. Dip 2019 [12] reported improved cystic duct–CBD junction visualization with fluorescence, both before dissection and after dissection; post-dissection visualization was 68.8% with near-infrared fluorescence versus 44.7% with white-light imaging. DOTIG 2025 [22] also showed that visualization improved after dissection, with cystic duct–CBD junction visualization increasing from 32.8% pre-dissection to 68.0% post-dissection (Table 2). Although Dip 2019 [12] demonstrated significantly improved visualization of several biliary structures with NIFC compared with white-light imaging before and after dissection, these anatomical findings should not be equated with improved clinical outcomes. Across the included trials, total operative time, conversion, length of stay, and postoperative complications generally showed no consistent improvement. Better visualization may facilitate anatomical orientation, but the available randomized evidence does not establish that it causes safer surgery or reduces biliary injury. Conversely, non-significant differences in uncommon complications do not establish equivalence, given the limited event counts.

**Figure 4:**
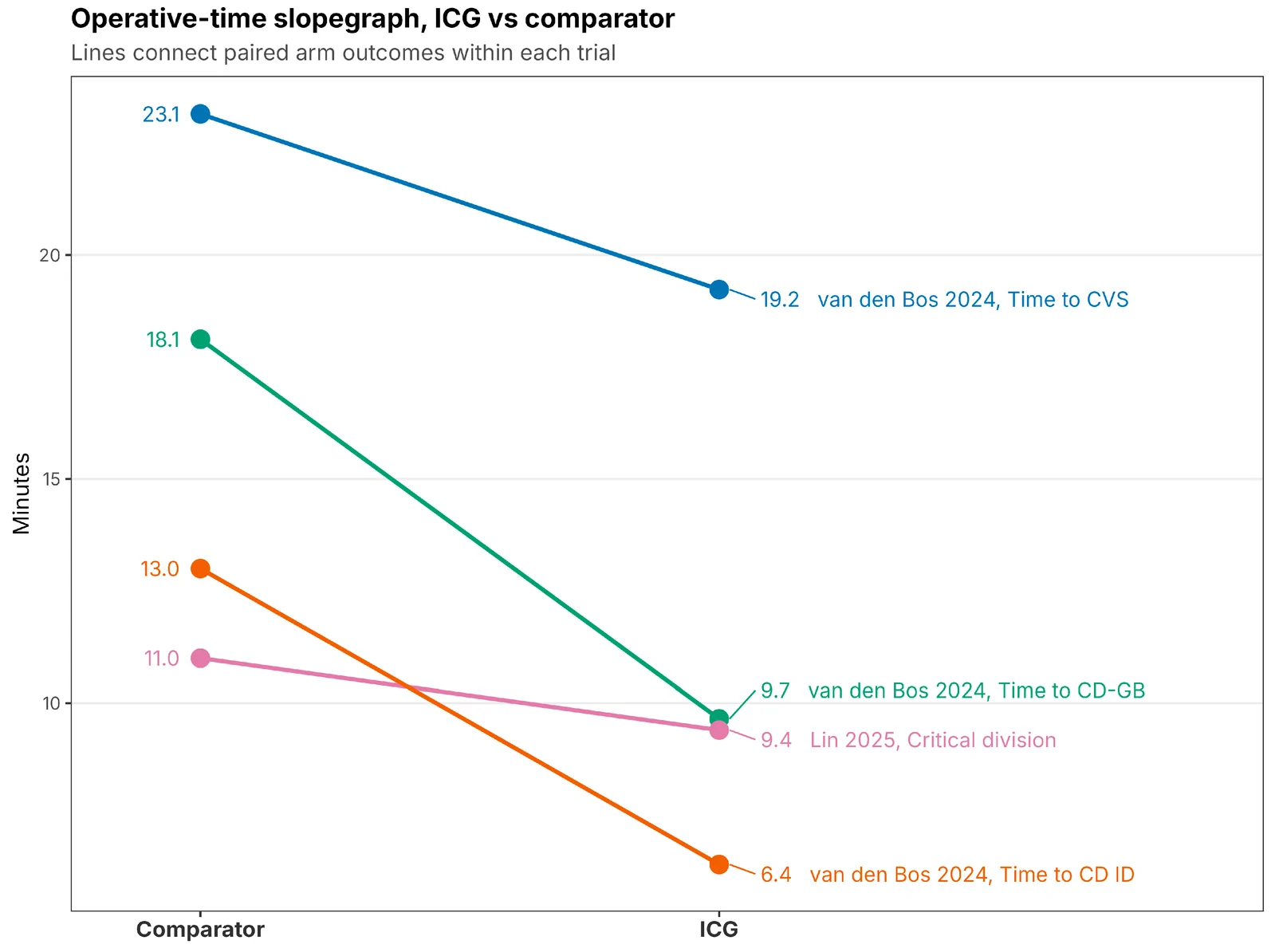
Operative-time slopegraph: ICG versus comparator [18,21]. Slopegraph of paired operative-time outcomes. Each line connects comparator and ICG values within the same study or endpoint.

Overall, several trials reported high cystic duct and CBD visualization rates with ICG fluorescence cholangiography, although these varied by anatomical structure, procedural phase, administration protocol, and patient population. Its clearest comparative advantage was over white-light imaging, particularly for pre-dissection identification of the CBD, CHD, and biliary junctions. Comparisons with radiographic IOC showed similar observed visualization rates for selected extrahepatic structures but did not consistently establish equivalence or noninferiority. IOC may also offer better proximal hepatic duct visualization: Lehrskov 2020 [13] reported right and left hepatic duct visualization in 27% of patients receiving ICG versus 85% receiving IOC (Table 1; Figure 4). These findings support ICG as an adjunct for real-time extrahepatic biliary visualization, while subjective assessment and risk of bias limit confidence in the reported advantages. They do not establish improved clinical outcomes or support replacing IOC when detailed proximal ductal mapping is required.

### 3.4. Operative Outcomes

Operative endpoints were heterogeneously reported and included cholangiography duration, time to identification of key structures, time to CVS, total operative time, conversion or bailout, and length of stay (Table 3). Across studies, the most consistent operative signal was that ICG shortened process-specific imaging or identification times, whereas effects on total operative time, conversion, and hospitalization were inconsistent or absent.

ICG-based imaging substantially reduced cholangiography or structure-identification time in several studies. In Symeonidis 2026 [10], cholangiography time was markedly shorter with IV and intracholecystic ICG than with IOC (110 ± 11.1 s and 125 ± 13.6 s vs. 305 ± 18.6 s; *p* < 0.001), although total operative time was unchanged across groups (46.5–47.3 min; *p* = 0.858). Similarly, Lehrskov 2020 [13] reported shorter cholangiography duration with ICG than with X-ray IOC (median 2.0 vs. 4.8 min; *p* < 0.001), with only a non-significant reduction in total operative time (45 vs. 54 min; *p* = 0.092). In van den Bos 2023 [18], NIRF accelerated cystic duct identification (6:24 vs. 13:00 min; *p* < 0.001), cystic duct–gallbladder transition identification (9:39 vs. 18:07 min; *p* < 0.001), and CVS achievement (19:14 vs. 23:09 min; *p* = 0.032), but did not significantly reduce total operative time (65:47 vs. 69:52 min; *p* = 0.198). These paired differences are summarized visually in Figure 4 and in Supplementary Figure S2. Defives 2025 [24] also found identical operative times with IOC and ICG in acute cholecystitis (82 vs. 82 min; *p* = 0.3), while Koong 2021 [14] found no difference in time to CVS (22.3 ± 12.9 vs. 22.8 ± 14.3 min; *p* = 0.867), and She 2022 [15] found no significant reduction in emergency LC operative time (149.5 vs. 140.5 min; *p* = 0.086). Ravikumar 2025 [25] was the main exception, reporting shorter operative time with ICG than non-ICG surgery (42.6 ± 5.2 vs. 48.3 ± 6.1 min; *p* = 0.05) and shorter length of stay (1.0 vs. 1.3 days; *p* = 0.045).

**Figure 5:**
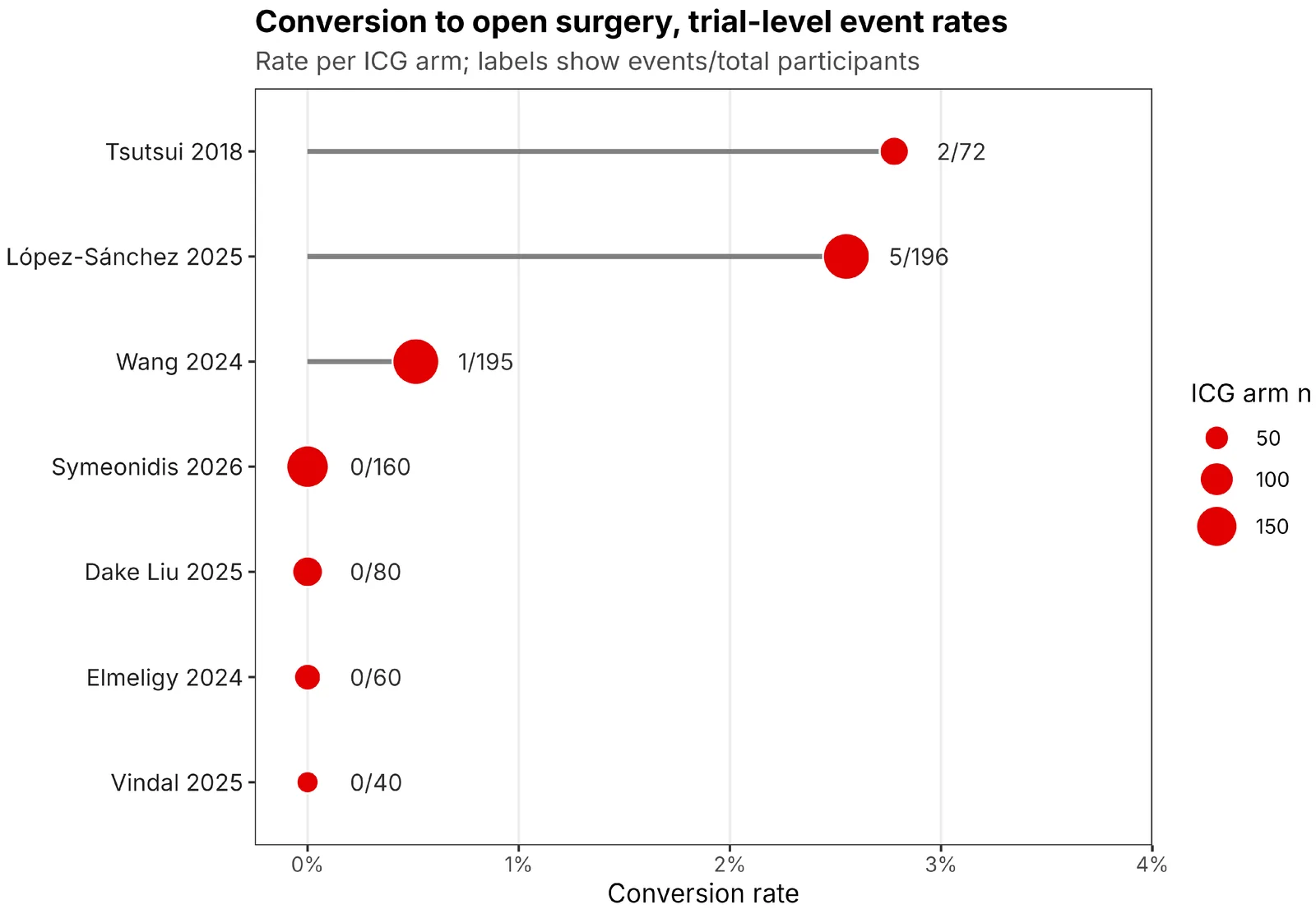
Conversion to open surgery: trial-level event rates [10,11,20,22,23,26,27]. Lollipop plot showing conversion-to-open event rates among ICG arms. Point labels show events over total participants. Point size reflects ICG arm sample size.

Conversion and bailout events were rare. No conversion occurred in the ICG arms of Symeonidis 2026, Elmeligy 2024, Vindal 2025, Lehrskov 2020, Dake Liu 2025, and Ravikumar 2025 [10,13,23,25,26], while low conversion rates were reported in van den Bos et al. [18], (3/143 vs. 1/151), Dip 2019 (1/321 vs. 4/318) [12], She 2022 (4/46 vs. 4/46) [15], Wang 2024 (1/195) [20], López-Sánchez 2025 (5/196) [22], and Tsutsui 2017 (2/72 across timing arms) [11]. The distribution of conversion events across ICG arms is shown in Figure 5.

Several trials reported shorter cholangiography duration or earlier identification of key biliary structures with ICG, suggesting potential improvements in specific components of intraoperative workflow. However, findings were inconsistent across populations and outcome definitions, and the available evidence did not establish reductions in total operative time, conversion rate, or length of stay. Any procedural time advantage may be influenced by inflammation, adhesions, dissection difficulty, surgeon experience, and institutional workflow, although the included trials did not establish how these factors modified the effect of ICG.

### 3.5. Safety Outcomes

Safety outcomes were infrequently observed across the included studies and were dominated by rare-event endpoints, including bile duct injury (BDI), bile leak, and ICG-related adverse events (Table 4). Overall, the available data did not suggest a consistent safety penalty from ICG use, although most studies were underpowered for uncommon complications.

Across the included studies, overall postoperative morbidity was generally similar between ICG and control groups. Symeonidis 2026 [10] reported low complication rates across IOC, IV ICG, and intracholecystic ICG groups (5.0%, 3.75%, and 2.5%, respectively; *p* = 0.573), with no delayed BDI, biliary stricture, or reintervention at 6 months. In VIFCAL [24], postoperative morbidity in grade I–II acute cholecystitis was also comparable between IOC and IV ICG: Clavien 0–II events occurred in 95% versus 94%, Clavien IIIa in 1.5% versus 0%, and Clavien IIIb in 2.9% versus 6%, with no mortality or ICG-related side effects. Similarly, She 2022 [15] found no significant difference in postoperative complications in emergency laparoscopic cholecystectomy (10.9% vs. 15.2%; *p* = 0.536), and Koong 2021 [14] reported comparable minor complications (2/30 vs. 2/33) with no major BDI (Table 4).

**Figure 6:**
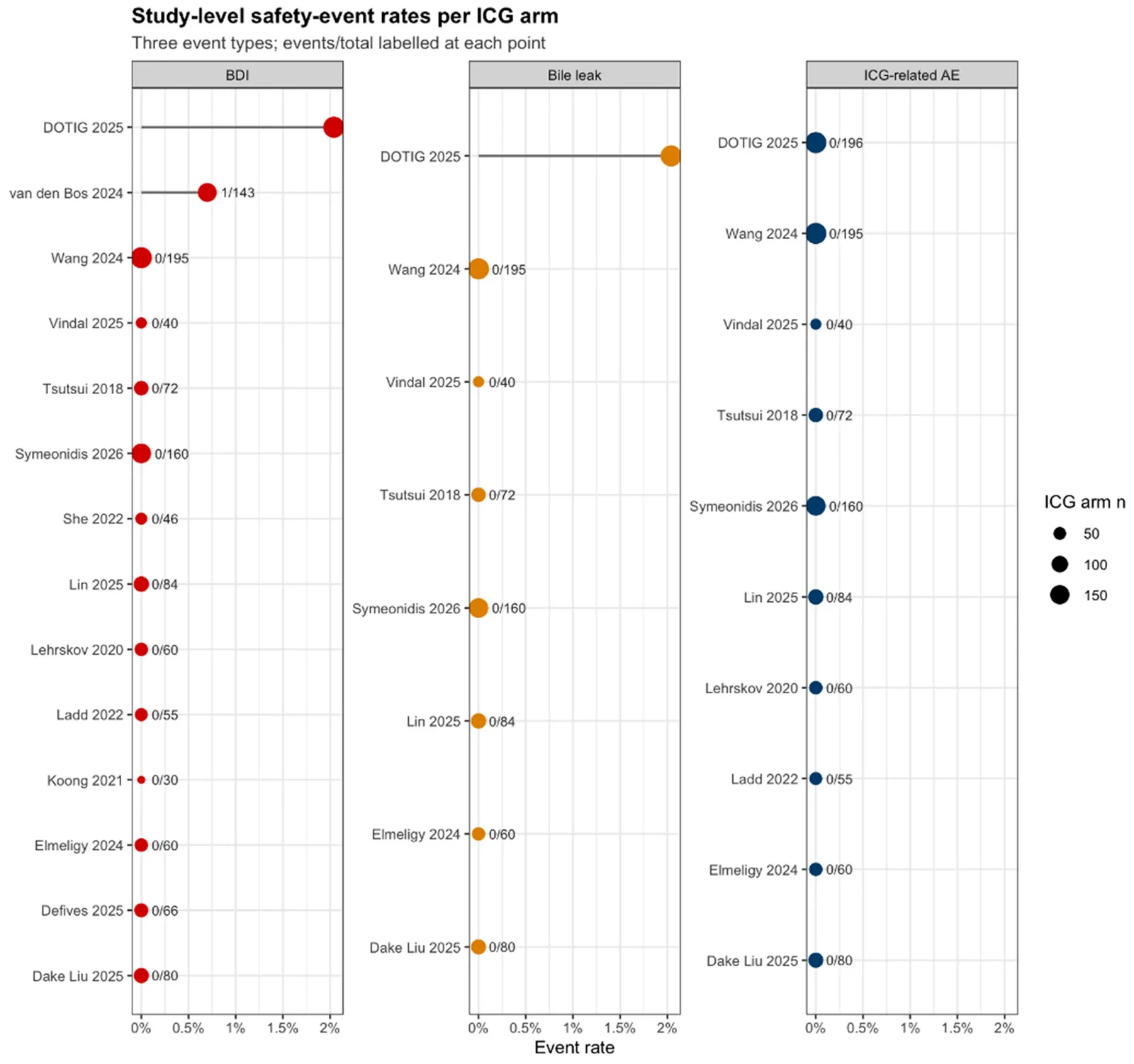
Study-level safety-event rates per ICG arm [10,11,13,14,15,17,18,20,21,22,23,24,26,27]. Lollipop plots showing study-level event rates for bile duct injury, bile leak, and ICG-related adverse events among ICG-treated arms. Point labels show events/total; point size reflects ICG arm sample size. BDI = bile duct injury; AE = adverse event.

Biliary injuries and bile leaks were rare. In the ICG arms, BDI was absent in most studies, including Symeonidis 2026, VIFCAL, Dip 2019, Koong 2021, Ravikumar 2025, Wang 2024, and the dose-finding trials [10,12,14,20,24,25]. The main exception was DOTIG 2025 [22], in which 4/196 patients had BDI, all Strasberg A, consisting of cystic-duct leaks managed with clip reinforcement or ERCP/stenting. Lehrskov 2020 [13] reported one accessory bile duct injury in the ICG group, while Dip 2019 [12] reported two BDIs in the white-light group and none in the NIFC group. Bile leak was also uncommon, reported in 2/143 versus 1/151 in FALCON [18], 1/46 versus 1/46 in She 2022 [15], and 0/84 versus 1/84 in Lin 2025 [21]. Study-level safety-event rates are shown in Figure 6.

ICG-specific adverse events were very rare. No ICG-related adverse event was reported in Symeonidis 2026, VIFCAL, Dip 2019, Tsutsui 2017, Wang 2024, Ladd 2022, or Dake Liu 2025 [10,11,12,17,20,23,24]. Elmeligy 2024 reported one delayed hypersensitivity reaction after IV ICG (1/30) [27], while FALCON reported one possible rash of unclear causality [18]. Vindal 2025 reported puncture-site ICG/bile spillage in 2/20 patients after intracholecystic injection, without clinical sequelae [26] (Supplementary Figure S3).

Taken together, bile duct injury and bile leak were uncommon, but the available sample sizes and event counts were insufficient to determine whether ICG reduces these complications. Low or zero event rates should not be interpreted as evidence of a protective effect, and non-significant differences do not establish equivalent safety. Similarly, improved anatomical visualization and shorter identification times have not established consistent reductions in conversion, postoperative morbidity, or hospitalization. Incomplete adverse-event reporting also limits conclusions about uncommon dye-related harms.

### 3.6. Route, Dose and Efficacy

**Figure 7:**
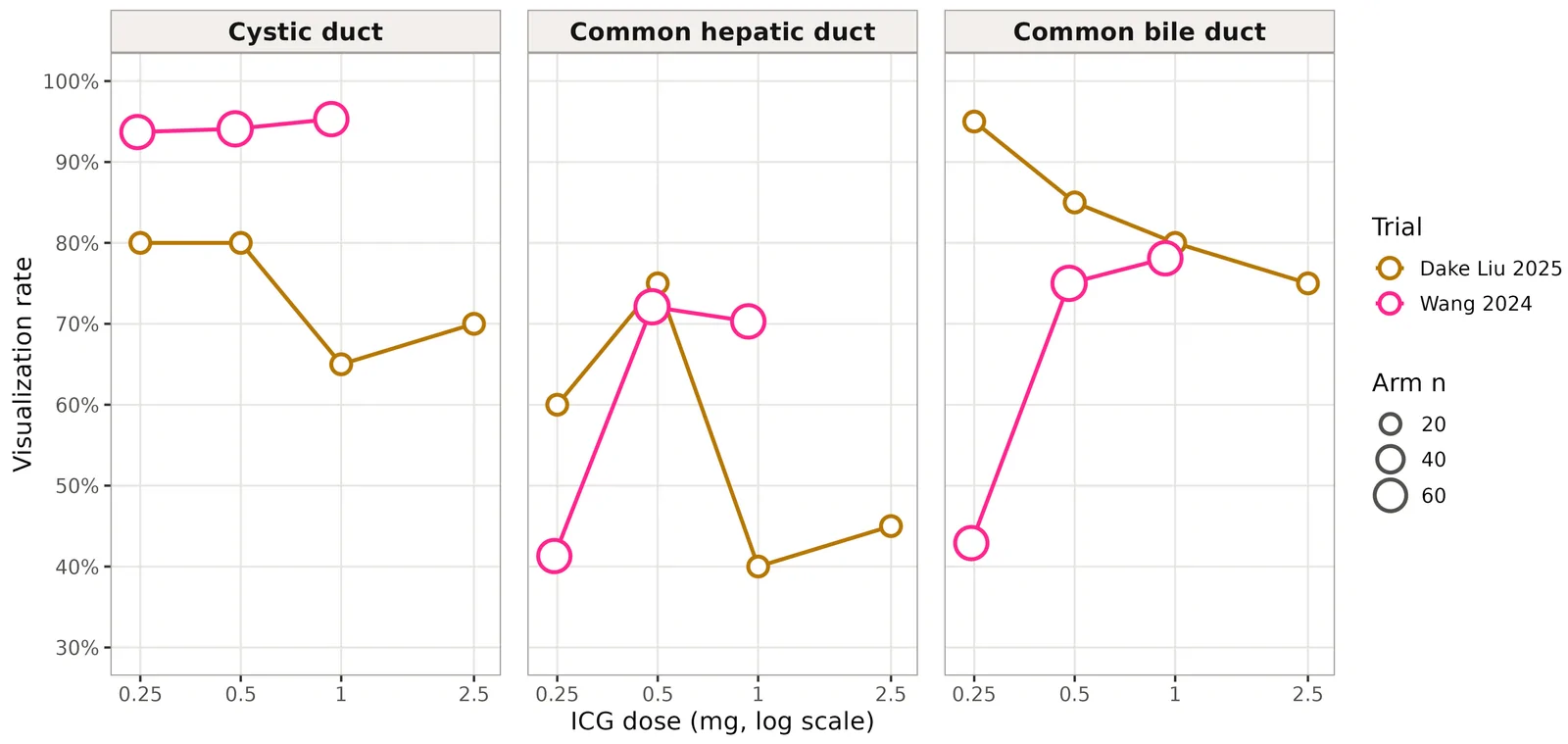
Within-trial connected-arm visualization versus ICG dose [20,23]. Connected-arm plots showing duct visualization rates across randomized ICG dose arms within dose-finding randomized trials. Each line connects arms from the same trial, reducing between-study confounding. Panels show cystic duct, common hepatic duct, and common bile duct visualization. Point size reflects arm sample size. The figure demonstrates that dose–response differs by duct and that higher doses do not uniformly improve visualization.

The efficacy of ICG fluorescence cholangiography was not determined by dose alone, but by the interaction between route of administration, hepatic background fluorescence, and timing from injection to incision. Across the available randomized dose-finding data, the clearest signal was that increasing ICG dose did not uniformly improve duct visualization. Instead, dose–response patterns differed by duct. Cystic duct visualization remained consistently high across most dose ranges, indicating that this structure is relatively easy to identify once fluorescence is present. In contrast, CHD and CBD visualization were more sensitive to protocol design, particularly when higher doses increased liver background fluorescence or when very low doses produced insufficient duct signal (Figure 7; Supplementary Figure S4).

In Dake Liu 2025 [23], CBD visualization decreased progressively with increasing fixed dose, from 95% at 0.25 mg to 75% at 2.5 mg. CHD visualization also peaked at lower doses, reaching 75% at 0.5 mg but falling to 40–45% at 1.0–2.5 mg. Conversely, in Wang 2024 [20], BMI-adjusted dosing showed an ascending pattern from 0.01 mg/BMI to 0.02–0.04 mg/BMI, especially for CHD and CBD visualization, suggesting that underdosing can also impair fluorescence performance (Figure 7). These findings support a biologically plausible therapeutic window rather than a simple “higher is better” dose–response relationship.

**Figure 8:**
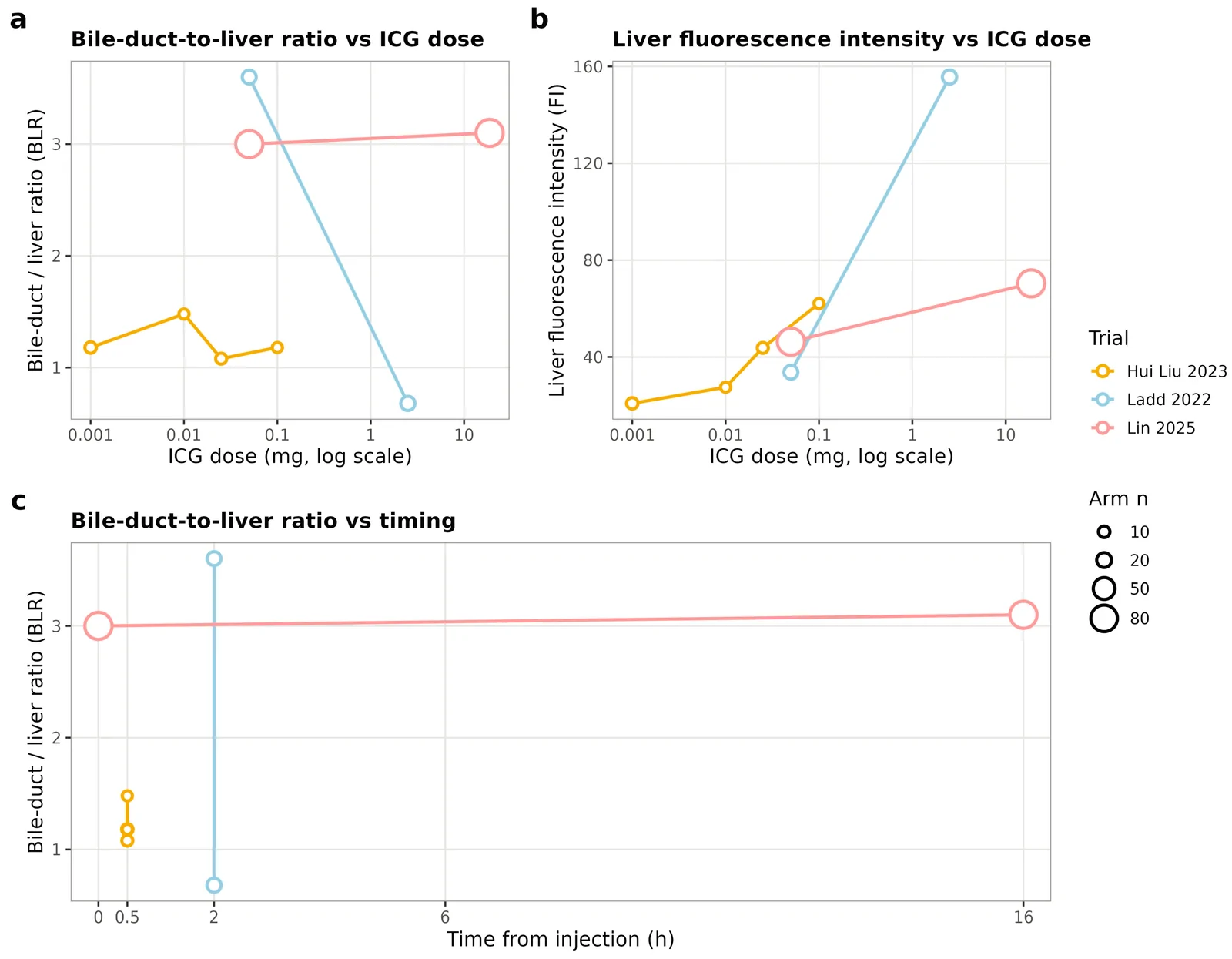
BLR and liver fluorescence versus dose and timing. Mechanistic plots of bile-duct-to-liver ratio and liver fluorescence intensity across dose-finding studies. Connected points represent arms within the same trial. BLR = bile-duct-to-liver fluorescence ratio; FI = fluorescence intensity. The figure shows that increasing ICG dose increases hepatic background fluorescence, while optimal duct visualization depends on maintaining duct-to-liver contrast.

The mechanistic data reinforce this interpretation. Liver fluorescence intensity increased with dose across Hui Liu 2023, Ladd 2022, and Lin 2025, while bile-duct-to-liver ratio (BLR) was highest when duct signal was preserved but liver background remained limited (Figure 8) [17,21]. The strongest mechanistic contrast came from Ladd 2022 [17], where 0.05 mg produced a BLR of 3.6 compared with 0.68 at 2.5 mg, while liver fluorescence rose from 33.7 to 155.6 arbitrary units at the standard dose. Similarly, Hui Liu 2023 [19] showed increasing liver fluorescence from 20.83 at 1 μg to 62.08 at 100 μg, while BLR remained modest and peaked at intermediate microdose levels. These data explain why excessive dose may degrade visualization of deeper or extra-cystic structures: the duct may fluoresce, but the liver background fluoresces more.

**Figure 9:**
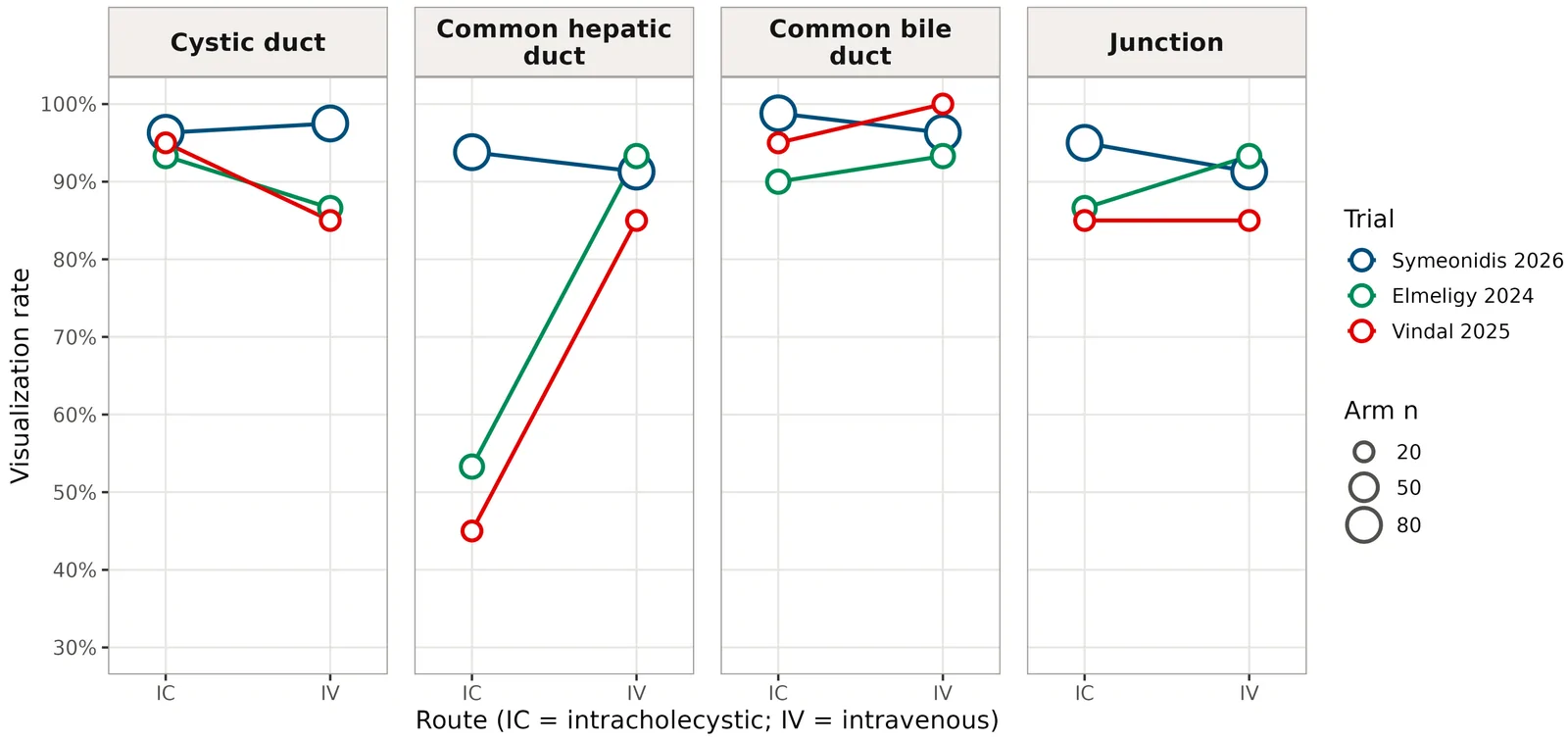
Route comparison: Intravenous versus intracholecystic ICG. Within-trial route comparison of IV and intracholecystic ICG across cystic duct, common hepatic duct, common bile duct, and junction visualization. Each line represents one trial, and point size reflects arm sample size. Intracholecystic ICG generally preserved cystic duct and CBD visualization while reducing liver fluorescence, but CHD visualization was more variable.

Route of administration produced a distinct pattern. Intracholecystic ICG reduced or eliminated liver fluorescence, but its performance depended on whether the target structure was adjacent to the gallbladder or required extra-gallbladder ductal distribution. Across Symeonidis 2026, Elmeligy 2024, and Vindal 2025, intracholecystic ICG generally preserved high cystic duct and CBD visualization, but CHD visualization was more variable and was lower than IV ICG in Elmeligy 2024 and Vindal 2025 [10,26,27]. In Vindal 2025, CHD visualization was 85% with IV ICG but only 45% with intracholecystic ICG, despite complete elimination of liver fluorescence in the intracholecystic arm [26]. This suggests that intracholecystic ICG may provide strong local biliary contrast but may not reliably opacify the proximal hepatic duct system (Figure 9).

Timing data were less definitive. Most CBD visualization arms clustered within a short preoperative window, approximately 0.5–1.5 h before incision, leaving limited information on delayed protocols (Supplementary Figure S5). Tsutsui 2017 suggested that delayed timing may improve duct-to-liver contrast, with CBD/liver ratio highest around 15 h and subjective Grade A visibility peaking at the same time point [11]. However, these data came from a fixed 25 mg protocol and cannot be directly extrapolated to modern low-dose strategies. Lin 2025 provided a more clinically relevant signal: despite a much higher dose in the preoperative arm, delayed timing was associated with lower liver background than expected, supporting the concept that hepatic clearance can partially offset high-dose liver fluorescence (Supplementary Figure S6) [21].

Randomized within-trial comparisons suggest that dose, timing, and administration route can influence fluorescence contrast under specific imaging and clinical conditions. However, the findings do not establish a universally optimal regimen. Fixed-dose, weight-based, BMI-based, and microdose protocols are not directly interchangeable, and comparisons that modify both dose and timing cannot isolate either component. Cross-study patterns should therefore be considered hypothesis-generating. Intracholecystic administration may reduce hepatic background fluorescence, but a consistent visualization advantage across biliary structures has not been demonstrated.

### 3.7. Clinical Heterogeneity and Narrative Synthesis by Patient Population

The 18 included studies differed substantially in operative urgency, disease acuity, previous biliary complications and procedural complexity. Six studies explicitly evaluated elective laparoscopic cholecystectomy [10,11,13,14,18,26], whereas two specifically investigated acute or emergency presentations [15,24]. Six included mixed clinical indications or operative settings [12,17,21,22,25,27]. In the remaining four studies, reporting was insufficient to establish a reliable elective-versus-emergency classification [16,19,20,23]. Consequently, elective scheduling, inflammatory disease and operative difficulty were considered separately rather than treating elective surgery as synonymous with uncomplicated disease. Tsutsui 2017 [11] excluded acute gallbladder inflammation, while the FALCON trial [18] excluded acute cholecystitis and restricted enrollment to ASA I–II patients. Conversely, Lehrskov 2020 [13] investigated elective surgery following complicated gallstone disease, including previous cholecystitis, pancreatitis or duct stones. Koong 2021 [14] also included substantial previously complicated disease and intraoperative difficulty; however, conversions and subtotal procedures were excluded from the primary timing analysis. Within this heterogeneous elective population, the FALCON trial [18] reported shorter time to achieve the critical view of safety, whereas Koong 2021 [14] found no significant difference. Lehrskov 2020 [13] and Symeonidis 2026 [10] reported similar observed visualization rates for the critical junction compared with radiographic cholangiography and shorter imaging-procedure duration, without a significant reduction in total operative duration. Evidence specifically addressing acute or emergency surgery was limited to two trials with different comparators. She 2022 [15] compared fluorescence-assisted surgery with conventional laparoscopic cholecystectomy in emergency acute cholecystitis and found no significant differences in conversion, operative duration, postoperative complications or hospital stay. Defives 2025 [24] evaluated acute cholecystitis using a conventional IOC strategy as the comparator and found no significant improvement in time to achieve the critical view of safety or total operative duration. Interpretation of the latter trial was complicated by post-randomization exclusions and frequent additional radiographic cholangiography in the fluorescence group. These findings did not establish a clinical advantage of fluorescence in acute surgery.

### 3.8. Risk-of-Bias and GRADE Assessment

**Figure 10:**
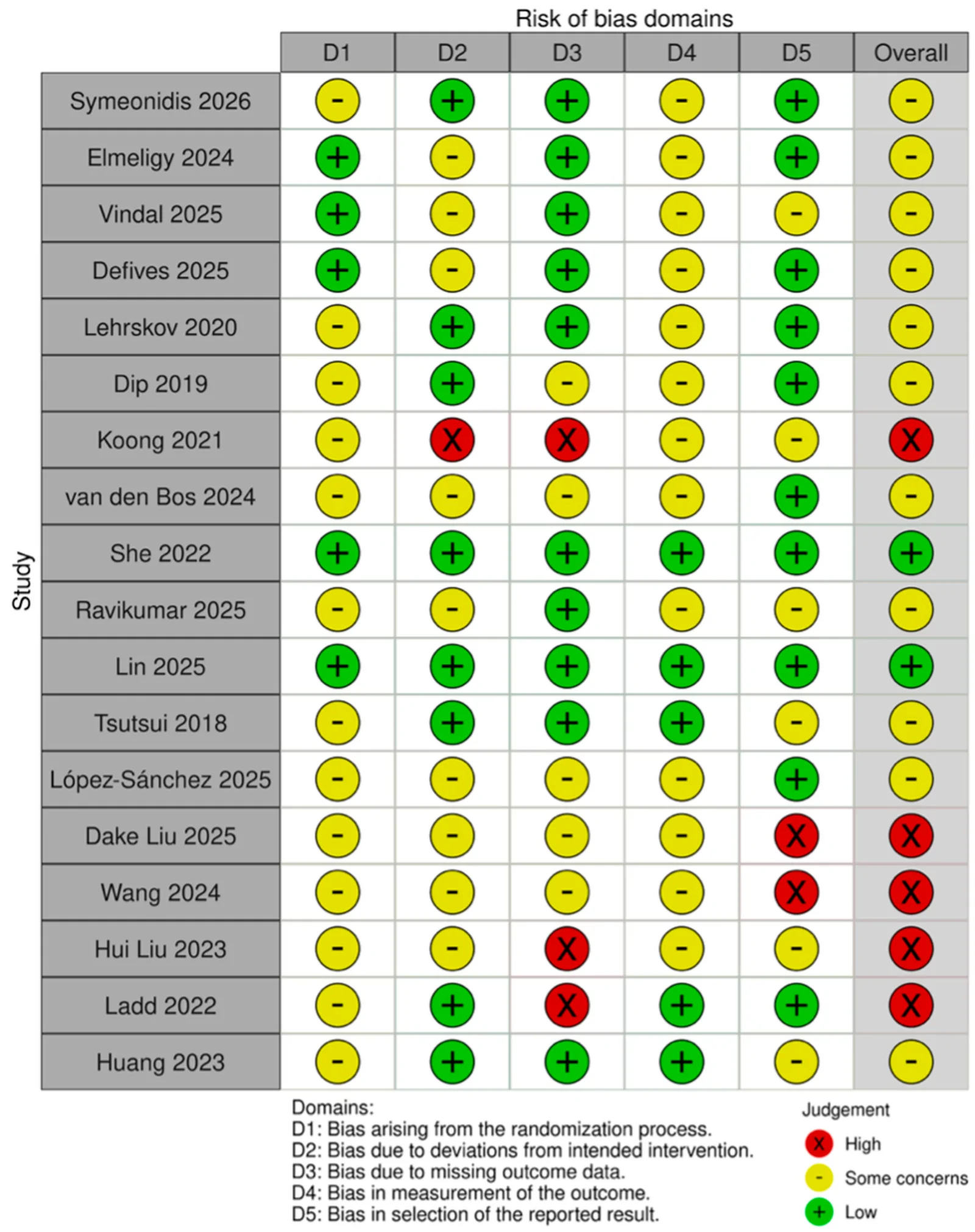
Risk-of-bias traffic light plot [10,11,12,13,14,15,16,17,18,19,20,21,22,23,24,25,26,27].

RoB 2 assessment (Figure 10) showed that most trials had some concerns, while only She et al., 2022, and Lin et al., 2025, were judged as low risk of bias overall. Five trials were rated high risk overall, mainly because of missing outcome data or selective reporting. The most common concern was outcome-measurement bias, which is important because fluorescence visualization is partly subjective and may depend on surgeon assessment, image quality, and blinding. These indicate that the findings of the descriptive synthesis should be interpreted cautiously, especially for subjective visualization endpoints and dose-optimization analyses. No funnel plot, asymmetry test, or trim-and-fill analysis was performed because the heterogeneous comparisons and outcome measures did not support a meaningful review-wide assessment. Consequently, the presence or extent of publication bias could not be determined.

Certainty of evidence varied by outcome and comparison. Moderate-certainty evidence supported improved pre-dissection visualization of the cystic duct, common hepatic duct and common bile duct with ICG fluorescence compared with white-light imaging, and shorter cholangiography procedure duration compared with radiographic IOC in elective surgery. Evidence concerning total operative duration and length of stay was of low certainty, while evidence concerning bile duct injury, conversion and postoperative morbidity was predominantly of very low certainty. Certainty for route, dose and timing comparisons was generally low or very low because of methodological limitations, small samples and incomplete or inconsistent reporting. These findings support anatomical visualization benefits but do not establish prevention of clinically important complications or a universally optimal administration regimen (Supplementary Figure S7).

## 4. Discussion

This systematic review found that ICG fluorescence cholangiography may assist biliary visualization during laparoscopic cholecystectomy, with its main benefit lying in improved or faster visualization of biliary anatomy rather than consistent improvement in operative outcomes or safety. Across comparative studies, ICG provided visualization rates comparable to conventional intraoperative cholangiography and generally superior to white-light imaging, particularly for pre- and post-dissection identification of the cystic duct, common hepatic duct, common bile duct, and biliary junctions. Several studies showed faster identification of key structures or shorter cholangiography time with ICG; however, total operative time, conversion, length of stay, and postoperative complications were usually unchanged. Bile duct injury, bile leak, conversion, and ICG-related adverse events were infrequently reported, but limited event counts and inconsistent reporting preclude firm conclusions regarding comparative safety or a protective effect. The clearest mechanistic signal was that fluorescence performance depends on protocol design: lower or optimized doses and appropriate timing may improve duct-to-liver contrast by reducing hepatic background fluorescence, whereas excessive dose or poorly timed administration may impair visualization.

Clinically, these findings suggest that ICG fluorescence cholangiography is best positioned as an anatomical guidance tool rather than as a replacement for surgical judgment or for conventional cholangiography in all circumstances. It may be particularly useful when early recognition of the CD, cystic duct–gallbladder junction, CBD, or variant anatomy could facilitate safe progression toward the CVS. However, conventional IOC retains advantages when detailed intrahepatic ductal mapping, stone detection, or definitive cholangiographic documentation is required. ICG and IOC should therefore be viewed as complementary rather than mutually exclusive technologies. Confidence in the reported visualization advantages is limited by the risk-of-bias profile of the included trials. Most studies had some concerns, and five were judged as high risk of bias. Surgeon-rated visualization and qualitative imaging outcomes are particularly susceptible to measurement bias when assessors are aware of the imaging technique. Missing outcome data and selective reporting further limit interpretation, particularly in several dose-finding trials. Accordingly, the observed advantages should be regarded as provisional rather than definitive evidence of comparative superiority.

The practical implication is that future implementation should focus less on whether ICG is used and more on how it is used. A standardized protocol should specify dose, timing, route, imaging platform, and target endpoint. Based on the available evidence, low-dose or BMI-adjusted intravenous protocols and carefully selected intracholecystic approaches deserve further evaluation. Future trials should report duct-specific visualization before and after dissection, time to key anatomical landmarks, liver fluorescence intensity, duct-to-liver contrast, surgeon confidence, conversion, bailout strategy, bile duct injury, bile leak, and ICG-specific adverse events. Without this level of reporting, protocol optimization will remain difficult.

The cost of ICG fluorescence cholangiography varies across Europe, the United States, and Asia according to equipment availability, institutional tariffs, and the components included in costing. European reports in two pediatric surgery centers describe approximately €40 per ICG vial and an initial investment of approximately €55,000 for a specific NIR imaging system and laparoscope [28]. In the United States, a published comparison reported costs of approximately US$14 for fluorescence cholangiography versus US$778 for conventional IOC [29], whereas a Singapore report cited subsidized patient charges of approximately US$180 versus US$50, respectively [30]. These estimates represent different costing approaches and should not be directly compared. Material, equipment, and professional-service costs should be distinguished from reimbursable charges, which depend on local payer arrangements. Although existing NIR equipment may limit additional expenditure, robust procedure-specific cost-effectiveness evidence remains limited [31].

This review has several strengths. It integrates evidence from randomized controlled trials across efficacy, operative, safety, and optimization outcomes. It separates duct-specific visualization endpoints rather than treating biliary visualization as a single homogeneous outcome. It also distinguishes between clinical visualization, operative efficiency, safety events, and mechanistic fluorescence metrics, allowing a more biologically coherent interpretation of why some protocols perform better than others. The limitations are also important, as the high heterogeneity between studies did not allow for the statistical analysis of the results and performing a meta-analysis. First, outcome definitions varied substantially across studies. “Visualization” could refer to pre-dissection detection, post-dissection confirmation, subjective grading, fluorescence intensity, bile-duct-to-liver ratio, or surgeon-rated usefulness. Second, ICG protocols differed widely in dose, route, timing, and imaging systems, limiting direct comparability. Third, several clinically important outcomes, including bile duct injury, conversion, bailout procedures, and ICG-related adverse events, were rare and inconsistently reported. Fourth, dose and timing data were sparse, and some apparent dose–response patterns may be confounded by trial design, case mix, or fluorescence platform. Another major limitation is the risk of bias directly affecting the validity of the primary visualization outcomes. Only two trials were judged at low risk overall, while most had some concerns and five were at high risk. Visualization depends on surgeon assessment, image quality, and assessor blinding, making these endpoints particularly vulnerable to outcome-measurement bias. Missing outcome data and selective reporting further reduce confidence in the reported findings. Consequently, the apparent visualization advantages should be interpreted cautiously and should not be regarded as definitive evidence of superiority. Finally, many studies were not powered for safety endpoints, and the absence of bile duct injury in most cohorts should not be interpreted as proof of injury prevention.

## 5. Conclusions

In conclusion, ICG fluorescence cholangiography may assist real-time anatomical orientation during laparoscopic cholecystectomy, with the clearest evidence supporting improved visualization of selected biliary structures compared with white-light imaging. However, subjective outcome assessment, risk of bias, and clinical heterogeneity limit confidence in the reported advantages. Improved visualization should not be equated with demonstrated clinical benefit, and current evidence is insufficient to establish comparative safety or reductions in bile duct injury, conversion, or postoperative morbidity. Randomized dose, timing, and route comparisons provide findings specific to the tested regimens and imaging systems, while cross-study observations remain hypothesis-generating and no universally optimal protocol has been established. Future adequately powered trials should evaluate clearly defined administration strategies using standardized duct-specific visualization measures and patient-important clinical outcomes.

## Figures and Tables

**Table 1:** Baseline characteristics of included randomized studies.

Study	Country	N Analyzed	Groups/Comparison	Age	Female Sex	BMI	Preoperative Diagnosis/Indication	ICG Protocol	Clinical Setting
Symeonidis 2026 [10]	Greece	240	IOC 80/IV ICG 80/IC ICG 80	56.1 ± 12.9/49.9 ± 10.7/51.3 ± 10.3 y	57/80, 71.25%/48/80, 60.0%/50/80, 62.5%	28.3 ± 5.82/27.9 ± 3.51/29.5 ± 2.33	Cholelithiasis: 76/80, 95%/77/80, 96.25%/77/80, 96.25%; adenomyomatosis: 3/80, 3.75%/1/80, 1.25%/3/80, 3.75%; polyps: 1/80, 1.25%/2/80, 2.5%/0/80	ICG VERDYE diluted to 2.5 mg/mL. IV route: 0.3 mg/kg 6 h before anesthesia. Intracholecystic route: 2.5 mL injected into the gallbladder under laparoscopic guidance using a 25-G needle; NIR imaging after ~3 min.	Elective LC
Elmeligy 2024 [27]	Egypt	60	IV ICG 30/transhepatic IC ICG 30	40.5 ± 11.5/43.2 ± 12.9 y	22/30, 73.3%/24/30, 80.0%	31.92 ± 3.94/28.48 ± 4.41	Biliary colic: 25/30, 83.3%/26/30, 86.6%; acute attack: 3/30, 10%/2/30, 6.6%; GB polyp: 1/30, 3.3%/2/30, 6.6%; gallstone pancreatitis: 1/30, 3.3%/0/30	IC ICG: 1.25 mg ICG diluted in saline to ~0.04 mg/mL; injected transhepatically into the gallbladder fundus using a Veress needle. Puncture site cauterized to reduce leakage.	LC, mixed benign biliary indications
Vindal 2025 [26]	India	40	IV ICG 20/IC ICG 20	37.90 ± 9.48/40.03 ± 12.85 y	16/20, 80%/16/20, 80%	NR	Gallstone disease; one empyema gallbladder excluded intraoperatively	IV ICG: 0.01 mg/kg administered 30–45 min before anesthesia after reconstitution in 5 mL distilled water. IC ICG: reconstituted dye diluted 1:10; 2 mL injected into the gallbladder lumen under vision using an 18-G spinal needle. Puncture site clipped to prevent leakage; gallbladder decompressed or Hartmann’s pouch stone displaced when required.	Elective LC for gallstone disease
Defives 2025 [24]	France	134	IOC 68/IV ICG 66	56/58 y	44/68, 65%/38/66, 58%	29/29	Grade I acute lithiasic cholecystitis: 32/68, 47%/40/66, 61%; Grade II: 36/68, 53%/26/66, 39%	IV ICG: Infracyanine^®^ reconstituted in 10 mL 5% glucose to 2.5 mg/mL; fixed 2.5 mg dose injected ≥30 min before intubation, up to 40 min in emergency cases. Control: standard IOC with IOMERON contrast.	Grade I–II acute lithiasic cholecystitis
Lehrskov 2020 [13]	Denmark	120	Fluorescence 60/X-ray IOC 60	52/58 y, median	32/60, 53.3%/45/60, 75.0%	27.4/29.0	Gallstone pancreatitis: 9/60, 15%/9/60, 15%; acute cholecystitis history: 37/60, 62%/26/60, 43%; cholangitis: 0/60/2/60, 3%; CBD stones: 14/60, 23%/23/60, 38%	IV ICG: Verdye^®^ 0.05 mg/kg, equivalent to 2.5–7.5 mg, injected immediately after anesthesia induction. NIR fluorescence imaging was used before Calot’s triangle dissection and during dissection at surgeon discretion to support CVS identification.	Elective LC
Dip 2019 [12]	Multicenter	639	WLI 318/NIFC 321	48.5 ± 15.5/48.3 ± 15.0 y	181/318, 57.1%/218/321, 67.9%	28.4 ± 6.4/27.5 ± 5.7	Cholelithiasis: 278/318, 87.7%/276/321, 86.5%; mild/moderate/severe inflammation broadly balanced; urgent/emergent cases included	IV ICG: 0.05 mg/kg injected ≥45 min preoperatively. NIFC performed before and after Calot’s triangle dissection, using alternating WLI and ICG fluorescence modes to identify extrahepatic biliary anatomy.	Elective, urgent, and emergent LC
Koong 2021 [14]	Malaysia	63	ICGFC-LC 30/LC 33	51 ± 13/51 ± 16 y	19/30, 63%/23/33, 70%	26.7 ± 5.4/25.9 ± 5.3	Biliary colic: 8/30, 27%/12/33, 36%; resolved acute cholecystitis: 10/30, 33%/9/33, 27%; pancreatitis: 3/30, 10%/1/33, 3%; ERCP-treated choledocholithiasis: 6/30, 20%/6/33, 18%; polyps: 2/30, 7%/2/33, 6%; empyema: 1/30, 3%/3/33, 9%	IV ICG: 2.5 mg IV bolus before anesthesia induction.	Elective LC
van den Bos 2023 [18]	Multicenter	294	NIRF-LC 143/CLC 151	53.8 ± 14.5/53.1 ± 15.6 y	87/143, 61.3%/97/151, 65.5%	27.6 ± 5.1/27.5 ± 5.1	Symptomatic cholecystolithiasis: 123/143, 85.9%/137/151, 90.7%; post-cholecystitis: 9/143, 6.3%/7/151, 4.6%; polyps: 4/143, 2.8%/4/151, 2.6%; post-biliary pancreatitis: 5/143, 3.5%/3/151, 2.0%	IV ICG: 2.5 mg injected ≥30 min before incision; repeat 2.5 mg dose after CVS in selected centers for combined biliary/arterial fluorescence. Control: standard CVS-based LC without IOC.	Elective LC
She 2022 [15]	Hong Kong	92	ICG 46/conventional 46	61.0, range 26–82/61.0, range 30–86 y	22/46, 47.8%/24/46, 52.2%	NR; body weight 65.5 kg in both groups	Acute cholecystitis; CBD stones on preoperative imaging: 15/46, 32.6%/11/46, 23.9%; preoperative ERCP: 18/46, 39.1%/15/46, 32.6%	IV ICG: 3.5 mg bolus ~30 min before anesthesia, followed by 10 mL saline flush. Additional 2–3 mL IV bolus used if vascular delineation was needed. Imaging with PINPOINT/SPY or OPAL1™ NIR/ICG systems.	Emergency LC
Ravikumar 2025 [25]	India	100	ICG 50/non-ICG 50	Most patients 40–50 y; exact group age NR	Overall 42/100, 42%	NR	Acute calculous cholecystitis: 10/50 ICG/18/50 non-ICG; acute-on-chronic cholecystitis: 9/50/7/50; chronically contracted GB: 5/50/3/50; symptomatic gallstone disease: 24/50/18/50; additional polyps/pancreatitis mainly non-ICG	IV ICG: 0.5 mL IV infusion 1 h preoperatively; standard four-port LC.	Mixed LC
Lin 2025 [21]	China	168	Intraoperative strategy 84/preoperative strategy 84	51.5, IQR 39.3–61.0/54.0, IQR 48.0–62.5 y	32/84, 38%/46/84, 55%	23.8, IQR 21.8–26.5/24.0, IQR 21.4–26.4	Acute cholecystitis: 2/84, 2.4%/4/84, 4.8%; gallbladder polyps: 4/84, 4.8%/5/84, 6.0%; chronic cholecystitis: 41/84, 48.8%/41/84, 48.8%; cholelithiasis: 37/84, 44.0%/34/84, 40.4%	IV ICG: PS group received 0.25 mg/kg 16 h preoperatively; IS group received 0.05 mg at anesthesia induction. Imaging performed with the PINPOINT fluorescence system.	LC, mostly elective
Tsutsui 2017 [11]	Japan	72	0, 3, 6, 9, 12, 15, 18, 24 h groups	63.0 ± 19.9/67.0 ± 14.6/58.0 ± 8.0/60.0 ± 10.6/58.0 ± 10.3/65.0 ± 8.9/72.0 ± 15.7/62.5 ± 15.0 y	Women: 2/9, 8/11, 6/9, 4/9, 2/9, 3/9, 1/8, 1/8	22.8 ± 3.5/21.0 ± 2.8/21.8 ± 4.2/24.3 ± 5.1/26.8 ± 1.8/23.7 ± 3.5/24.0 ± 3.1/23.6 ± 2.4	Mostly cholecystolithiasis; also gallbladder polyps, adenomyomatosis, chronic cholecystitis	IV ICG: fixed 25 mg dose administered at randomized timing intervals: 0, 3, 6, 9, 12, 15, 18, or 24 h before surgery.	Elective LC
López-Sánchez 2025 [22]	Spain	196 analyzed	G1 47/G2 45/G3 44 randomized, 43 evaluable/G4 60	Overall median 64 y, range 21–91; group medians 66/60/69/61 y	Overall 121/196, 61.7%; by group 33/47, 25/45, 25/44, 38/60	Overall 27.9; groups 30.5/27.1/28.9/28.4	Biliary colic 71/196, 36.2%; cholecystitis 41/196, 20.9%; pancreatitis 39/196, 19.9%; cholangitis 24/196, 12.2%; choledocholithiasis 13/196, 6.6%; polyps 6/196, 3.1%; adenomyomatosis 2/196, 1%	IV ICG: Verdye^®^ diluted to 2.5 mg/mL. Four protocols: 2.5 mg >3 h preop; 2.5 mg 15–30 min preop; 0.05 mg/kg >3 h preop; 0.05 mg/kg 15–30 min preop; G3/G4 mean dose 3.8 ± 0.9 mg.	Mostly elective LC
Dake Liu 2025 [23]	China	80	0.25 mg 20/0.50 mg 20/1.00 mg 20/2.50 mg 20	55.80 ± 11.93/61.20 ± 12.16/62.45 ± 15.00/55.85 ± 15.70 y	13/20, 65%/13/20, 65%/11/20, 55%/9/20, 45%	28.80 ± 4.04/27.10 ± 3.70/26.99 ± 4.20/25.95 ± 4.51	Gallstones with chronic cholecystitis: 11/20, 55%/7/20, 35%/8/20, 40%/9/20, 45%; acute cholecystitis: 7/20, 35%/12/20, 60%/11/20, 55%/10/20, 50%; polyps or stones with polyps in small numbers	IV ICG: 1 mL of the assigned ICG solution injected via peripheral vein 0.5–3 h preoperatively. Fluorescence and white-light modes were alternated intraoperatively to guide Calot’s triangle dissection and visualize the cystic duct, common hepatic duct, and cystic artery.	LC
Wang 2024 [20]	China	195	0.01 mg/BMI 63/0.02 mg/BMI 68/0.04 mg/BMI 64	53.7 ± 11.9/51.5 ± 14.3/52.0 ± 13.3 y	40/63, 63.5%/39/68, 57.4%/39/64, 60.4%	24.51 ± 3.6/24.03 ± 3.0/23.34 ± 3.7	Cholelithiasis: 58/63, 92.1%/65/68, 95.6%/57/64, 89.1%; fatty liver: 12/63, 19.0%/11/68, 16.2%/13/64, 20.3%	IV ICG: 1 mL assigned dose injected 0.5–3 h preoperatively. Randomized groups: 0.01, 0.02, or 0.04 mg/BMI. Fluorescence/white-light imaging alternated during Calot’s triangle dissection.	Fluorescent LC
Hui Liu 2023 [19]	China	33 analyzed	1 µg 10/10 µg 7/25 µg 9/100 µg 7	53.5 ± 17.7/64.6 ± 17.9/57.7 ± 11.7/55.6 ± 13.1 y	4/10, 40%/6/7, 85.7%/6/9, 66.7%/5/7, 71.4%	24.62 ± 1.98/24.466 ± 2.11/25.22 ± 2.28/25.13 ± 2.42	Cholecystolithiasis and cholecystitis in all analyzed patients; LC or LC + CBDE	IV ICG: randomized dose of 1, 10, 25, or 100 µg injected 30 min preoperatively after anesthesia induction. Fluorescence assessed at three timepoints: before dissection, before cystic duct clipping, and before closure/CBD exploration.	LC with ultra-high-definition 4K fluorescent system
Ladd 2022 [17]	USA	55 randomized; 35 quantitative	Low-dose 29/standard-dose 26	53.0, IQR 18.0/60.0, IQR 23.5 y	20/29, 69.0%/20/26, 76.9%	30.1, IQR 9.4/27.7, IQR 5.7	Cholelithiasis: 23/29, 79.3%/17/26, 65.4%; acute cholecystitis: 1/29, 3.5%/1/26, 3.8%; chronic cholecystitis: 1/29, 3.5%/2/26, 7.7%; polyps: 2/29, 6.9%/2/26, 7.7%; other biliary indications in small numbers	0.05 mg vs. 2.5 mg IV ICG, preoperatively on day of surgery.	LC
Huang 2022 [16]	China	40	0.025 mg 10/0.1 mg 10/0.25 mg 10/2.5 mg 10	57.7 ± 4.987/51.6 ± 4.492/64.4 ± 4.738/55.7 ± 5.325 y	5/10, 50%/2/10, 20%/6/10, 60%/7/10, 70%	22.82 ± 0.733/24.41 ± 1.220/23.89 ± 1.132/23.57 ± 1.353	Cholecystitis in all groups; cholecystolithiasis: 3/10/5/10/8/10/7/10; cholecystic polyps: 3/10/3/10/2/10/2/10	IV ICG: randomized dose of 0.025, 0.1, 0.25, or 2.5 mg injected 30 min preoperatively after anesthesia induction. NIRF imaging was recorded before dissection, before cystic duct clipping, and before closure/CBD exploration.	LC with real-time fluorescent cholangiography

Abbreviations: BMI, body mass index; CBD, common bile duct; CBDE, common bile duct exploration; CLC, conventional laparoscopic cholecystectomy; CVS, critical view of safety; GB, gallbladder; IC, intracholecystic; ICG, indocyanine green; ICGFC, indocyanine green fluorescence cholangiography; IOC, intraoperative cholangiography; IV, intravenous; LC, laparoscopic cholecystectomy; NIFC/NIRF, near-infrared fluorescence cholangiography/imaging; NR, not reported in extracted results; WLI, white-light imaging.

**Table 2:** Visualization of biliary anatomy across included studies.

Study	Comparison	N Analyzed	Cystic Duct Visualization	Common Hepatic Duct Visualization	Common Bile Duct Visualization	Junction/Other Biliary Structures	Fluorescence Contrast/Qualitative Visualization	Main Visualization Finding
Symeonidis 2026 [10]	IOC vs. IV ICG vs. IC ICG	80/80/80	72/77, 93.5% vs. 78/80, 97.5% vs. 77/80, 96.25%	70/77, 90.9% vs. 73/80, 91.25% vs. 75/80, 93.75%	71/77, 92.2% vs. 77/80, 96.25% vs. 79/80, 98.75%	Critical junction: 71/77, 92.2% vs. 73/80, 91.25% vs. 76/80, 95.0%	Surgeon satisfaction: 2.9 ± 0.79 vs. 4.1 ± 1.05 vs. 4.0 ± 0.91	IV and IC ICG achieved visualization comparable to IOC, with no failed ICG investigations
Elmeligy 2024 [27]	IV ICG vs. transhepatic IC ICG	30/30	Pre: 20/30, 66.6% vs. 22/30, 73.3%; post: 26/30, 86.6% vs. 28/30, 93.3%	Pre: 16/30, 53.3% vs. 14/30, 46.6%; post: 28/30, 93.3% vs. 16/30, 53.3%	Pre: 20/30, 66.6% vs. 15/30, 50%; post: 28/30, 93.3% vs. 27/30, 90%	Cystic–hepatic junction pre: 16/30, 53.3% vs. 14/30, 46.6%; post: 28/30, 93.3% vs. 26/30, 86.6%	Liver fluorescence: 30/30, 100% vs. 2/30, 6.6%	IC reduced liver fluorescence and improved CD visualization; IV better visualized CHD after dissection
Vindal 2025 [26]	IV ICG vs. IC ICG	20/20	Pre: 14/20, 70% vs. 17/20, 85%; post: 17/20, 85% vs. 19/20, 95%	17/20, 85% vs. 9/20, 45%	20/20, 100% vs. 19/20, 95%	CD-CHD confluence: 17/20, 85% vs. 17/20, 85%	Liver fluorescence: 20/20, 100% vs. 0/20, 0%; visualization score 4.25 ± 1.17 vs. 4.1 ± 1.18	IC produced faster visualization and no liver fluorescence; IV better visualized CHD
Defives 2025 [24]	IOC vs. IV ICG	68/66	Pre: 19/68, 28% vs. 19/66, 29%; post: 61/68, 90% vs. 57/66, 86%	NR	34/68, 50% vs. 37/66, 56%	NR	NR	No meaningful visualization advantage of ICG over IOC in grade I–II acute cholecystitis
Lehrskov 2020 [13]	IV ICG vs. X-ray IOC	60/60	54/60, 90% vs. 51/60, 85%	52/60, 87% vs. 51/60, 85%	56/60, 93% vs. 51/60, 85%	Critical junction: 49/60, 82% vs. 51/60, 85%; RHD/LHD: 16/60, 27% vs. 51/60, 85%	Ease score: 1.90 ± 0.89 vs. 2.36 ± 1.03	ICG comparable for CD/CHD/CBD but inferior for right and left hepatic ducts
Dip 2019 [12]	NIFC vs. WLI	321/318	Pre: 213/320, 66.6% vs. 113/312, 36.2%; post: 311/321, 96.9% vs. 309/318, 97.2%	Pre: 92/318, 28.9% vs. 34/312, 10.9%; post: 168/321, 52.3% vs. 97/318, 30.5%	Pre: 157/318, 49.4% vs. 64/311, 20.6%; post: 243/321, 75.7% vs. 159/318, 50.0%	Cystic–CBD junction pre: 96/319, 30.1% vs. 31/311, 10.0%; post: 220/320, 68.8% vs. 142/318, 44.7%; accessory ducts post: 20/321, 6.2% vs. 6/318, 1.9%	Surgeon perception favored NIFC; >80% reported increased safety/confidence/use preference	NIFC significantly improved pre-dissection and post-dissection visualization of most structures compared with WLI
Koong 2021 [14]	ICGFC-LC vs. LC	30/33	NR	NR	NR	NR	NR	Duct-specific visualization rates not reported; time to CVS assessed
van den Bos 2023 [18]	NIRF-LC vs. CLC	143/151	Time to CD identification: 6:24 vs. 13:00 min	NR	NR	Time to CD–GB transition: 9:39 vs. 18:07 min; time to CVS: 19:14 vs. 23:09 min	Difficulty score 1.9 vs. 2.0	NIRF accelerated identification of key structures and CVS achievement
She 2022 [15]	ICG vs. conventional emergency LC	46/46	NR	NR	NR	NR	Fluorescence Grade A 18/46, 39.1%; Grade B 13/46, 28.3%; Grade C 14/46, 30.4%	Qualitative fluorescence was feasible in emergency LC; 56.5% of surgeons reported benefit
Ravikumar 2025 [25]	ICG vs. non-ICG	50/50	NR	NR	NR	NR	NR	No duct-specific visualization rates reported
Lin 2025 [21]	Intraoperative vs. preoperative ICG	84/84	Score: 4.1 ± 0.7 vs. 4.2 ± 0.8	Score: 4.1 ± 0.7 vs. 3.7 ± 0.6	Score: 4.0 ± 0.7 vs. 4.0 ± 0.7	NR	CBD/liver SBR: 3.0 ± 0.8 vs. 3.1 ± 1.2; liver FI: 46.3 ± 12.9 vs. 70.4 ± 26.2	Intraoperative strategy maintained CBD/liver contrast while reducing liver background and improving CHD score
Tsutsui 2017 [11]	0–24 h timing groups	72	CD/liver ratio exceeded 1 at 12–24 h	NR	CBD/liver ratio highest at 15 h	GB/liver ratio improved at delayed timing	Subjective Grade A visibility peaked at 15 h	Delayed preoperative timing improved duct-to-liver contrast, with best visibility around 15 h
López-Sánchez 2025 [22]	Four dose/timing groups	195 evaluable for visualization	Overall pre: 50.7%; post: 80.0%	Overall pre: 36.4%; post: 55.3%	Overall pre: 61.0%; post: 78.4%	CD-CBD junction: 32.8% to 68.0%; CD-GB junction: 43.5% to 75.3%; anatomical variations: 2.0% to 9.7%	HELPFUL: 40% very helpful; DISTURBED score differed significantly across groups	Visualization improved substantially after dissection; timing >3 h improved selected outcomes more than dose
Dake Liu 2025 [23]	0.25 vs. 0.50 vs. 1.00 vs. 2.50 mg	20/20/20/20	16/20, 80%/16/20, 80%/13/20, 65%/14/20, 70%	12/20, 60%/15/20, 75%/8/20, 40%/9/20, 45%	19/20, 95%/17/20, 85%/16/20, 80%/15/20, 75%	NR	Excellent imaging: 18/20, 90%/15/20, 75%/12/20, 60%/10/20, 50%; fluorescence contrast worsened with dose	Lower fixed doses produced better subjective imaging and faster bile duct visualization
Wang 2024 [20]	0.01 vs. 0.02 vs. 0.04 mg/BMI	63/68/64	59/63, 93.7%/64/68, 94.1%/61/64, 95.3%	26/63, 41.3%/49/68, 72.1%/45/64, 70.3%	27/63, 42.9%/51/68, 75.0%/50/64, 78.1%	Cystic–CBD junction: 30/63, 47.6%/51/68, 75.0%/51/64, 79.7%; cystic–GB junction: 53/63, 84.1%/61/68, 89.7%/59/64, 92.2%	Favorable observation: 25/63, 39.7%/49/68, 72.1%/45/64, 70.3%	0.02 and 0.04 mg/BMI improved CHD, CBD, and junction visualization compared with 0.01 mg/BMI
Hui Liu 2023 [19]	1 vs. 10 vs. 25 vs. 100 µg	10/7/9/7	NR	NR	Qualitative CBD fluorescence described	NR	Before dissection: liver FI 20.83/27.50/43.75/62.08; bile duct FI 22.22/38.89/46.91/74.07; BLR 1.18/1.48/1.08/1.18	10–25 µg appeared to balance duct fluorescence and liver background; 1 µg insufficient and 100 µg excessive
Ladd 2022 [17]	0.05 mg vs. 2.5 mg	29/26; quantitative 17/18	Score: 3.6 ± 1.3 vs. 3.5 ± 1.2	Score: 3.6 ± 1.4 vs. 2.8 ± 1.3	Score: 3.6 ± 1.3 vs. 3.1 ± 1.3	Aberrant ducts: 2.8 ± 1.3 vs. 2.3 ± 0.4	Bile duct/liver ratio: 3.6 vs. 0.68; bile duct/background fat ratio: 7.5 vs. 3.3; liver FI: 33.7 vs. 155.6	Low-dose ICG markedly improved duct-to-liver and duct-to-background contrast by reducing liver fluorescence
Huang 2022 [16]	0.025 vs. 0.1 vs. 0.25 vs. 2.5 mg	10/10/10/10	NR	NR	CBD FI and BLR measured	NR	BLR before dissection highest with 0.1 mg; BLR ≥1 before dissection: 7/10, 8/10, 3/10, 0/10; BLR ≥ 3 before dissection: 1/10, 4/10, 0/10, 0/10	0.1 mg within 30 min preoperatively provided the best duct-to-liver contrast; 2.5 mg produced excessive liver background

Abbreviations: BLR, bile duct-to-liver ratio; CBD, common bile duct; CD, cystic duct; CHD, common hepatic duct; CVS, critical view of safety; FI, fluorescence intensity; GB, gallbladder; IC, intracholecystic; ICG, indocyanine green; IOC, intraoperative cholangiography; IV, intravenous; NIFC/NIRF, near-infrared fluorescence cholangiography/imaging; NR, not reported; RHD/LHD, right/left hepatic duct; SBR, signal-to-background ratio; WLI, white-light imaging.

**Table 3:** Operative outcomes across included studies.

Study	Comparison	N Analyzed	Imaging/Cholangiography Time	Time to CVS or Key Structure Identification	Total Operative Time	Conversion/Bailout	Length of Stay
Symeonidis 2026 [10]	IOC vs. IV ICG vs. IC ICG	80/80/80	Cholangiography time: 305 ± 18.6 vs 110 ± 11.1 vs 125 ± 13.6 s, *p* < 0.001	NR	47.1 ± 7.31 vs. 46.5 ± 7.43 vs. 47.3 ± 6.86 min, *p* = 0.858	Failed IOC: 3/80; converted to open; IV/IC completed in all	NR
Elmeligy 2024 [27]	IV ICG vs. transhepatic IC ICG	30/30	NR	NR	32 ± 16 vs. 38 ±2 3 min, *p* = 0.140	NR	1 vs. 1 day
Vindal 2025 [26]	IV ICG vs. IC ICG	20/20	NR	CD pre-dissection: 33.05 ± 10.58 vs. 3.18 ± 2.09 min, *p* < 0.0001; CHD: 34.17 ± 8.99 vs. 12.11 ± 10.59 min, *p* < 0.05; CD-CHD confluence: 34.15 ± 8.94 vs. 7.37 ± 8.63 min, *p* < 0.01; CBD: 35.50 ± 8.88 vs. 6.15 ± 7.76 min, *p* < 0.005	73.7 ± 18.11 vs. 74.45 ± 12.57 min, *p* = 0.882	NR	NR
Defives 2025 [24]	IOC vs. IV ICG	68/66	Optimal cystic duct visualization: 22 vs. 26 min, *p* = 0.3	Time to CVS: 30 vs. 32 min, *p* = 0.4	82 vs. 82 min, *p* = 0.3	Conversion: 1/68 vs. 0/66	3 vs. 3 days, *p* = 0.7
Lehrskov 2020 [13]	IV ICG vs. X-ray IOC	60/60	Cholangiography duration: 2.0 min, range 0.5–5.0 vs. 4.8 min, range 1.3–17.6, *p* < 0.001	NR	45 min, range 25–138 vs. 54 min, range 33–163, *p* = 0.092	Conversion: 0/60 vs. 1/60	NR
Dip 2019 [12]	NIFC vs. WLI	321/318	NR	NR	NR	Open procedure: 1/321 vs. 4/318	NR
Koong 2021 [14]	ICGFC-LC vs. LC	30/33	NR	Time to CVS: 22.3 ± 12.9 vs. 22.8 ± 14.3 min, *p* = 0.867	NR	Conversions excluded before final analysis: 2 vs. 3	NR
van den Bos 2023 [18]	NIRF-LC vs. CLC	143/151	NR	Time to CD identification: 6:24 vs. 13:00 min, *p* < 0.001; CD–GB transition: 9:39 vs. 18:07 min, *p* < 0.001; CA–GB transition: 15:06 vs. 19:35 min, *p* = 0.011; time to CVS: 19:14 vs. 23:09 min, *p* = 0.032	65:47 vs. 69:52 min, *p* = 0.198	Conversion: 3 vs. 1, *p* = 0.285	1 vs. 1 night, *p* = 0.055
She 2022 [15]	ICG vs. conventional emergency LC	46/46	Median ICG injection-to-surgery interval: 67 min, range 16–1150	NR	149.5 min, range 75–260 vs. 140.5 min, range 60–285, *p* = 0.086	Conversion: 4/46 vs. 4/46, *p* = 1.000	4.0 days, range 1–21 vs. 3.5 days, range 2–25, *p* = 0.380
Ravikumar 2025 [25]	ICG vs. non-ICG	50/50	NR	NR	42.6 ± 5.2 vs. 48.3 ± 6.1 min, *p* = 0.05	Conversion: 0 vs. 0; bailout: 0 vs. 0	1 day, range 1–2 vs. 1.3 days, range 1–2, *p* = 0.045
Lin 2025 [21]	Intraoperative vs. preoperative ICG	84/84	NR	Critical division action time: 9.4 min, IQR 7.8–10.5 vs. 11.0 min, IQR 8.8–11.9, *p* < 0.001	NR	NR	NR
Tsutsui 2017 [11]	0, 3, 6, 9, 12, 15, 18, 24 h timing groups	72	NR	NR	98.0 ± 31.7/90.0 ± 29.6/71.0 ± 35.1/62.0 ± 22.3/85.0 ± 30.0/80.0 ± 32.0/99.5 ± 29.0/69.0 ± 27.8 min	Open conversion: 0/0/1/0/0/0/1/0	3.0 ± 0.3/ 3.0 ± 0.6/ 3.0 ± 3.3/ 3.0 ± 0.4/ 3.0 ± 0.3/ 3.0 ± 0.3/ 3.0 ± 1.4/ 3.0 ± 2.6
López-Sánchez 2025 [22]	Four dose/timing groups	196 analyzed	NR	NR	Overall median 60 min, range 25–187; G1 60, G2 55, G3 60, G4 60 min; NS	Conversion: 5/196, 2.6%	Overall median 1 day, range 1–17; NS across groups
Dake Liu 2025 [23]	0.25 vs. 0.50 vs. 1.00 vs. 2.50 mg	20/20/20/20	Time to bile duct visualization: 15.44 ± 3.25/18.28 ± 2.70/21.13 ± 5.22/22.91 ± 5.85 min, *p* < 0.001	NR	NR	Open conversion: 0 in all groups	4/4/4/4 days, *p* = 0.492
Wang 2024 [20]	0.01 vs. 0.02 vs. 0.04 mg/BMI	63/68/64	NR	NR	Mean operative time overall 46 ± 17 min	Conversion: 1/63 vs. 0/68 vs. 0/64	NR
Hui Liu 2023 [19]	1 vs. 10 vs. 25 vs. 100 µg	10/7/9/7	Fluorescence assessed before dissection, before clipping, and before closure	NR	110.1 ± 39.4/115.0 ± 26.3/82.4 ± 27.6/84.4 ± 25.6 min, *p* = 0.12	NR	3.7 ± 2.4/5.4 ± 4.8/3.1 ± 1.4/3.4 ± 0.9 days, *p* = 0.43
Ladd 2022 [17]	0.05 mg vs. 2.5 mg	29/26	NR	NR	NR	NR	NR
Huang 2022 [16]	0.025 vs. 0.1 vs. 0.25 vs. 2.5 mg	10/10/10/10	ICG injection to surgery: 19.43 ± 3.50/21.30 ± 1.78/14.86 ± 3.42/24.20 ± 4.21 min, *p* = 0.149	Surgery to triangle dissection: 34.43 ± 2.72/38.50 ± 2.26/33.57 ± 2.64/41.20 ± 6.43 min, *p* = 0.693; surgery to clipping: 52.29 ± 6.14/55.40 ± 3.19/73.71 ± 14.09/65.00 ± 8.88 min, *p* = 0.401	71.43 ± 7.01/82.30 ± 6.47/96.43 ± 15.34/82.40 ± 9.05 min, *p* = 0.546	NR	2.43 ± 0.20/2.30 ± 0.15/3.29 ± 0.47/2.70 ± 0.45 days, *p* = 0.217

Abbreviations: CA, cystic artery; CBD, common bile duct; CD, cystic duct; CLC, conventional laparoscopic cholecystectomy; CVS, critical view of safety; GB, gallbladder; IC, intracholecystic; ICG, indocyanine green; ICGFC, indocyanine green fluorescence cholangiography; IOC, intraoperative cholangiography; IV, intravenous; LC, laparoscopic cholecystectomy; NIFC/NIRF, near-infrared fluorescence cholangiography/imaging; NR, not reported; NS, not significant; WLI, white-light imaging.

**Table 4:** Safety outcomes across included studies.

Study	Comparison	N Analyzed	Overall Complications	Bile Duct Injury/Biliary Injury	Bile Leak/Biliary Complications	Conversion/Bailout	ICG-Related Adverse Events	Main Safety Finding
Symeonidis 2026 [10]	IOC vs. IV ICG vs. IC ICG	80/80/80	4/80, 5.0% vs. 3/80, 3.75% vs. 2/80, 2.5%, *p* = 0.573	0 vs. 0 vs. 0	No delayed bile duct injury, biliary stricture, or reintervention at 6 months	3/80 IOC; 0 IV/IC	0 reported	Low complication rates across groups; no biliary safety signal with either ICG route
Elmeligy 2024 [27]	IV ICG vs. transhepatic IC ICG	30/30	Intraoperative: 0 vs. 0; postoperative: 0 vs. 0	0 vs. 0	NR	NR	Delayed hypersensitivity: 1/30 IV vs. 0/30 IC	Both routes were safe; transhepatic IC avoided bile/ICG spillage
Vindal 2025 [26]	IV ICG vs. IC ICG	20/20	0 vs. 2	0 vs. 0	NR	NR	0 reported	IC puncture-site spillage occurred in 2/20, 10%, without clinical consequence
Defives 2025 [24]	IOC vs. IV ICG	68/66	Clavien 0–II: 65/68, 95% vs. 62/66, 94%; Clavien IIIa: 1/68, 1.5% vs. 0/66; IIIb: 2/68, 2.9% vs. 4/66, 6%	0 vs. 0	Biliary complications: 2/68 vs. 0/66	Conversion: 1/68 vs. 0/66	0 vs. 0 side effects	In acute cholecystitis, ICG did not increase morbidity or dye-related adverse events; reoperation: 1.5% vs. 0%; readmission: 4.4% vs. 3.0%; mortality 0
Lehrskov 2020 [13]	IV ICG vs. X-ray IOC	60/60	Postoperative complications: 0 vs. 1 mild pancreatitis	Accessory bile duct injury: 1/60 vs. 0/60	NR	Conversion: 0/60 vs. 1/60	NR	One accessory duct injury occurred after fluorescence cholangiography; no broad safety excess identified; IOC group: 1 mild pancreatitis; CBD stones detected in 2 IOC patients
Dip 2019 [12]	NIFC vs. WLI	321/318	Overall complication count NR	BDI: 0/321 vs. 2/318	NR	Open procedure: 1/321 vs. 4/318	0/321	Both bile duct injuries occurred in the WLI group; no ICG adverse reactions
Koong 2021 [14]	ICGFC-LC vs. LC	30/33	Minor complications: 2/30 vs. 2/33	Major BDI: 0 vs. 0	NR	Conversions excluded before final analysis: 2 vs. 3	One IV catheter-site extravasation excluded before analysis	No major biliary injury; minor complications were comparable
van den Bos 2023 [18]	NIRF-LC vs. CLC	143/151	Complications: 17, 12.8% vs. 9, 6.4%, *p* = 0.126	NR	Bile leak: 2 vs. 1	Conversion: 3 vs. 1, *p* = 0.285	Possible rash: 1/143, causality unclear	No statistically significant difference in complication rate; one possible dye/anesthetic-related rash; Clavien IIIa: 2 vs. 1; IIIb: 4 vs. 2; LOS 1 vs. 1 night
She 2022 [15]	ICG vs. conventional emergency LC	46/46	Postoperative complications: 5/46, 10.9% vs. 7/46, 15.2%, *p* = 0.536	NR	Bile leak: 1/46 vs. 1/46	Conversion: 4/46 vs. 4/46, *p* = 1.000	NR	In emergency LC, ICG did not increase complications, bile leak, reoperation, or conversion
Ravikumar 2025 [25]	ICG vs. non-ICG	50/50	Excessive bleeding >100 mL: 4/50 vs. 2/50, *p* = 0.48	0 vs. 0	0 vs. 0	Conversion: 0 vs. 0; bailout: 0 vs. 0	NR	No bile duct injury, bowel injury, bile leak, conversion, or bailout intervention
Lin 2025 [21]	Intraoperative vs. preoperative ICG	84/84	Major complications NR	NR	Minor bile leak: 0/84 vs. 1/84	NR	NR	One minor bile leak occurred in the preoperative group and resolved after drainage
Tsutsui 2017 [11]	0–24 h timing groups	72	Intraoperative complications: 0 in all groups; postoperative complications: 0/0/1/0/0/0/0/1	NR	NR	Open conversion: 0/0/1/0/0/0/1/0	0/72	No adverse events due to ICG; timing groups did not differ in surgical safety outcomes
López-Sánchez 2025 [22]	Four dose/timing groups	196	Intraoperative complications: 99/196, 50.5%; postoperative complications: 49/196, 25%	BDI: 4/196, 2%, all Strasberg A	Two intraoperative CD leaks treated with clip reinforcement; two postoperative leaks treated with ERCP/stent	Conversion: 5/196, 2.6%	No ICG-specific adverse event reported in extracted results	BDI events were low-grade Strasberg A and managed intraoperatively or endoscopically; mortality 0
Dake Liu 2025 [23]	0.25 vs. 0.50 vs. 1.00 vs. 2.50 mg	20/20/20/20	Detailed complication rates NR	NR	NR	Open conversion: 0 in all groups	NR	Postoperative recovery parameters were similar across dose groups
Wang 2024 [20]	0.01 vs. 0.02 vs. 0.04 mg/BMI	63/68/64	No dose-specific complication increase reported	0 in all groups	NR	Conversion: 1/63 vs. 0/68 vs. 0/64	0/195	No adverse reactions to ICG and no bile duct injuries across BMI-based dose groups
Hui Liu 2023 [19]	1 vs. 10 vs. 25 vs. 100 µg	10/7/9/7	NR	NR	NR	NR	NR	No significant differences in operative time or hospitalization across microdose groups; detailed adverse-event data not reported
Ladd 2022 [17]	0.05 mg vs. 2.5 mg	29/26	NR	NR	NR	NR	0/55	No harms or unintended effects occurred in either low-dose or standard-dose group
Huang 2022 [16]	0.025 vs. 0.1 vs. 0.25 vs. 2.5 mg	10/10/10/10	NR	NR	NR	NR	NR	No significant differences in blood loss, postoperative hospitalization, operative time, or liver tests across dose groups

Abbreviations: BDI, bile duct injury; CD, cystic duct; ERCP, endoscopic retrograde cholangiopancreatography; IC, intracholecystic; ICG, indocyanine green; ICGFC, indocyanine green fluorescence cholangiography; IOC, intraoperative cholangiography; IV, intravenous; LC, laparoscopic cholecystectomy; NIFC/NIRF, near-infrared fluorescence cholangiography/imaging; NR, not reported; WLI, white-light imaging.

## Data Availability

No new data were created or analyzed in this study.

## Supplementary Materials

The following supporting information can be downloaded at https://www.mdpi.com/article/10.3390/medicina62102022/s1: Table S1: PRISMA 2020 Checklist.; Table S2: Search Strategy; Figure S1: Paired duct-visualization rates for ICG versus comparator; Figure S2: Operative-time outcomes: ICG versus comparator;. Figure S3: Crude pooled rare-event rates across reporting trials;. Figure S4: Dose-response: CBD visualization across arms;. Figure S5: Timing-response: CBD visualization versus injection-to-incision interval;. Figure S6: Arm-level operative time versus ICG dose;. Figure S7: GRADE assessment of certainty of evidence for ICG fluorescence cholangiography during laparoscopic cholecystectomy.
