# Supplementary material for: Optimizing Indocyanine Green Fluorescence Cholangiography in Laparoscopic Cholecystectomy: A Systematic Review of Randomized Controlled Trials on Dose, Timing, Route, Efficacy, and Safety
Source: Medicina (Kaunas). 2026 Oct 19;62(10):2022. doi: 10.3390/medicina62102022 (PMC13707089; doi:10.3390/medicina62102022)
Supplement: Supplementary file 1 [file medicina-62-02022-s001.zip › medicina-4572855-supplementary.pdf]

Supplementary Materials

Supplementary Table S1: PRISMA 2020 Checklist.

Section and Topic	Item #	Checklist item	Location where item is reported
**TITLE**			
Title	1	Identify the report as a systematic review.	P1
**ABSTRACT**			
Abstract	2	See the PRISMA 2020 for Abstracts checklist.	P1
**INTRODUCTION**			
Rationale	3	Describe the rationale for the review in the context of existing knowledge.	P2
Objectives	4	Provide an explicit statement of the objective(s) or question(s) the review addresses.	P2
**METHODS**			
Eligibility criteria	5	Specify the inclusion and exclusion criteria for the review and how studies were grouped for the syntheses.	P3
Information sources	6	Specify all databases, registers, websites, organisations, reference lists and other sources searched or consulted to identify studies. Specify the date when each source was last searched or consulted.	P3-4
Search strategy	7	Present the full search strategies for all databases, registers and websites, including any filters and limits used.	P3-4
Selection process	8	Specify the methods used to decide whether a study met the inclusion criteria of the review, including how many reviewers screened each record and each report retrieved, whether they worked independently, and if applicable, details of automation tools used in the process.	P3-4
Data collection process	9	Specify the methods used to collect data from reports, including how many reviewers collected data from each report, whether they worked independently, any processes for obtaining or confirming data from study investigators, and if applicable, details of automation tools used in the process.	P3-4
Data items	10a	List and define all outcomes for which data were sought. Specify whether all results that were compatible with each outcome domain in each study were sought (e.g. for all measures, time points, analyses), and if not, the methods used to decide which results to collect.	P3-4
	10b	List and define all other variables for which data were sought (e.g. participant and intervention characteristics, funding sources). Describe any assumptions made about any missing or unclear information.	P3-4
Study risk of bias assessment	11	Specify the methods used to assess risk of bias in the included studies, including details of the tool(s) used, how many reviewers assessed each study and whether they worked independently, and if applicable, details of automation tools used in the process.	P4
Effect measures	12	Specify for each outcome the effect measure(s) (e.g. risk ratio, mean difference) used in the synthesis or presentation of results.	NA
Synthesis methods	13a	Describe the processes used to decide which studies were eligible for each synthesis (e.g. tabulating the study intervention characteristics and comparing against the planned groups for each synthesis (item #5)).	NA
	13b	Describe any methods required to prepare the data for presentation or synthesis, such as handling of missing summary statistics, or data conversions.	NA
	13c	Describe any methods used to tabulate or visually display results of individual studies and syntheses.	NA
	13d	Describe any methods used to synthesize results and provide a rationale for the choice(s). If meta-analysis was performed, describe the model(s), method(s) to identify the presence and extent of statistical heterogeneity, and software package(s) used.	NA
	13e	Describe any methods used to explore possible causes of heterogeneity among study results (e.g. subgroup analysis, meta-regression).	NA
	13f	Describe any sensitivity analyses conducted to assess robustness of the synthesized results.	NA
Reporting bias assessment	14	Describe any methods used to assess risk of bias due to missing results in a synthesis (arising from reporting biases).	NA

Section and Topic	Item #	Checklist item	Location where item is reported
Certainty assessment	15	Describe any methods used to assess certainty (or confidence) in the body of evidence for an outcome.	P4
**RESULTS**			
Study selection	16a	Describe the results of the search and selection process, from the number of records identified in the search to the number of studies included in the review, ideally using a flow diagram.	P4-5
	16b	Cite studies that might appear to meet the inclusion criteria, but which were excluded, and explain why they were excluded.	P5-6
Study characteristics	17	Cite each included study and present its characteristics.	P5-13
Risk of bias in studies	18	Present assessments of risk of bias for each included study.	P31-32
Results of individual studies	19	For all outcomes, present, for each study: (a) summary statistics for each group (where appropriate) and (b) an effect estimate and its precision (e.g. confidence/credible interval), ideally using structured tables or plots.	P13-31
Results of syntheses	20a	For each synthesis, briefly summarise the characteristics and risk of bias among contributing studies.	NA
	20b	Present results of all statistical syntheses conducted. If meta-analysis was done, present for each the summary estimate and its precision (e.g. confidence/credible interval) and measures of statistical heterogeneity. If comparing groups, describe the direction of the effect.	NA
	20c	Present results of all investigations of possible causes of heterogeneity among study results.	NA
	20d	Present results of all sensitivity analyses conducted to assess the robustness of the synthesized results.	NA
Reporting biases	21	Present assessments of risk of bias due to missing results (arising from reporting biases) for each synthesis assessed.	NA
Certainty of evidence	22	Present assessments of certainty (or confidence) in the body of evidence for each outcome assessed.	Suppl.fig. 7
**DISCUSSION**			
Discussion	23a	Provide a general interpretation of the results in the context of other evidence.	P33
	23b	Discuss any limitations of the evidence included in the review.	P34
	23c	Discuss any limitations of the review processes used.	P34
	23d	Discuss implications of the results for practice, policy, and future research.	P33-34
**OTHER INFORMATION**			
Registration and protocol	24a	Provide registration information for the review, including register name and registration number, or state that the review was not registered.	P2
	24b	Indicate where the review protocol can be accessed, or state that a protocol was not prepared.	P2
	24c	Describe and explain any amendments to information provided at registration or in the protocol.	P2-3
Support	25	Describe sources of financial or non-financial support for the review, and the role of the funders or sponsors in the review.	P35
Competing interests	26	Declare any competing interests of review authors.	P35
Availability of data, code and other materials	27	Report which of the following are publicly available and where they can be found: template data collection forms; data extracted from included studies; data used for all analyses; analytic code; any other materials used in the review.	P35

Supplementary Table S2. Search Strategy

**Pubmed**	(indocyanine green OR ICG OR near infrared OR near-infrared OR near infrared fluorescence OR near-infrared fluorescence OR fluorescence imaging OR fluorescent imaging OR fluorescent cholangiography OR near-infrared fluorescence cholangiography OR NIRF OR NIFC) AND (laparoscopic cholecystectomy OR laparoscopic cholecystectomies OR cholecystectomy OR cholecystectomies) AND (biliary anatomy OR biliary structure OR biliary structures OR bile duct OR bile ducts OR common bile duct OR common hepatic duct OR cystic duct OR Calot triangle OR cystohepatic triangle OR cholangiography OR visualization OR visualisation OR identification)
**Scopus**	(<br>"indocyanine green" OR ICG OR "near infrared" OR near-infrared OR NIRF OR "fluorescence cholangiography" OR "fluorescent cholangiography" OR "fluorescence imaging"<br>)<br>AND<br>(<br>cholecystectomy OR "laparoscopic cholecystectomy" OR gallbladder OR cholecystitis OR cholelithiasis<br>)<br>AND<br>(<br>"bile duct" OR "bile ducts" OR "common bile duct" OR CBD OR "common hepatic duct" OR CHD OR "cystic duct" OR "Calot triangle" OR "Calot's triangle" OR "critical view of safety" OR CVS OR cholangiography OR visualization OR visualisation OR identification<br>)<br>AND<br>(<br>random* OR trial OR comparative OR comparison OR control OR conventional OR "white light" OR "intraoperative cholangiography" OR IOC OR dose OR timing OR route OR intravenous OR intracholecystic<br>)
**CENTRAL**	(cholecystectom* OR cholecystitis OR cholelithiasis OR gallbladder) AND ("indocyanine green" OR ICG OR fluorescen* OR "near infrared" OR "near-infrared" OR NIFC OR NIRF)

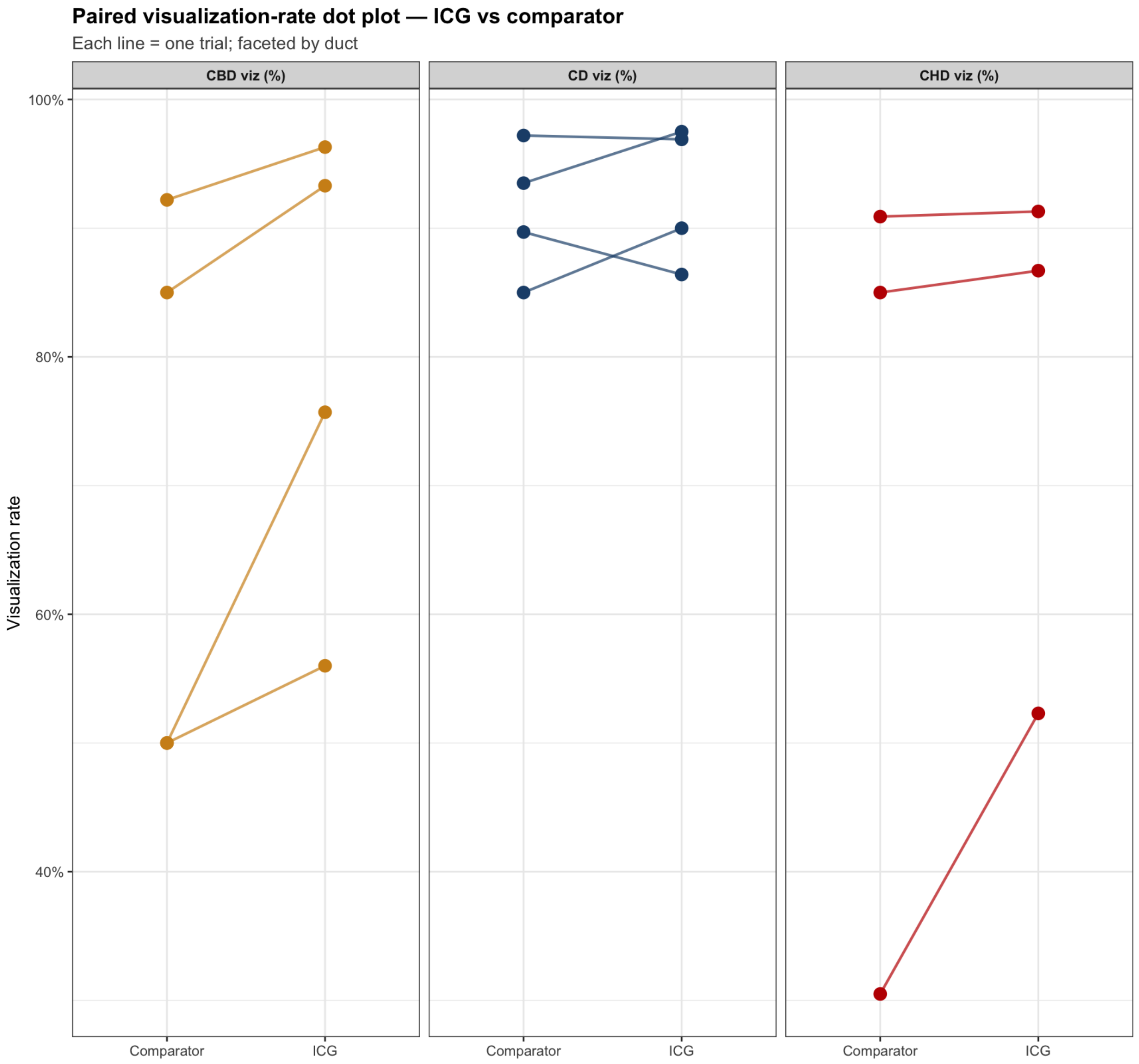

Supplementary Figure S1. Paired duct-visualization rates for ICG versus comparator. Each line represents one paired comparison within the same study, with the left point showing the comparator arm and the right point showing the ICG arm. Panels show common bile duct (CBD), cystic duct (CD), and common hepatic duct (CHD) visualization rates. The plot includes studies with both an ICG and comparator arm and

available duct-specific visualization data: Symeonidis 2026, Defives 2025, Lehrskov 2020, and Dip 2019 for CBD and CD visualization, and Symeonidis 2026, Lehrskov 2020, and Dip 2019 for CHD visualization. Defives 2025 was not included in the CHD panel because CHD visualization was not reported. Comparators included IOC, X-ray IOC, and white-light imaging, depending on the original study.

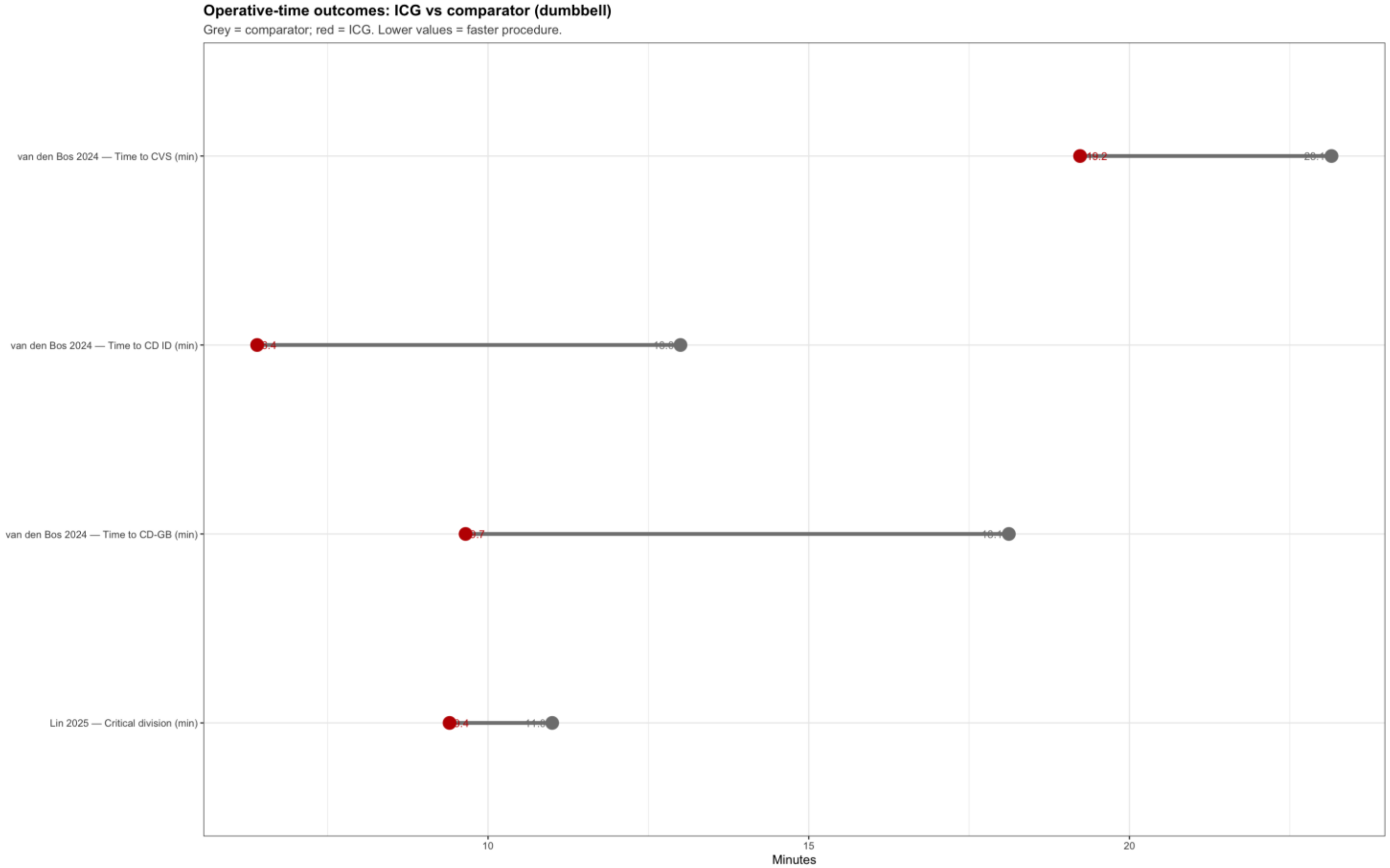

Supplementary Figure S2. Operative-time outcomes: ICG versus comparator. Dumbbell plot of paired operative-time outcomes from studies reporting ICG and comparator arms. Red points represent ICG; grey points represent comparator. Lower values indicate faster identification or shorter operative time. Lines connect paired outcomes within the same study or endpoint.

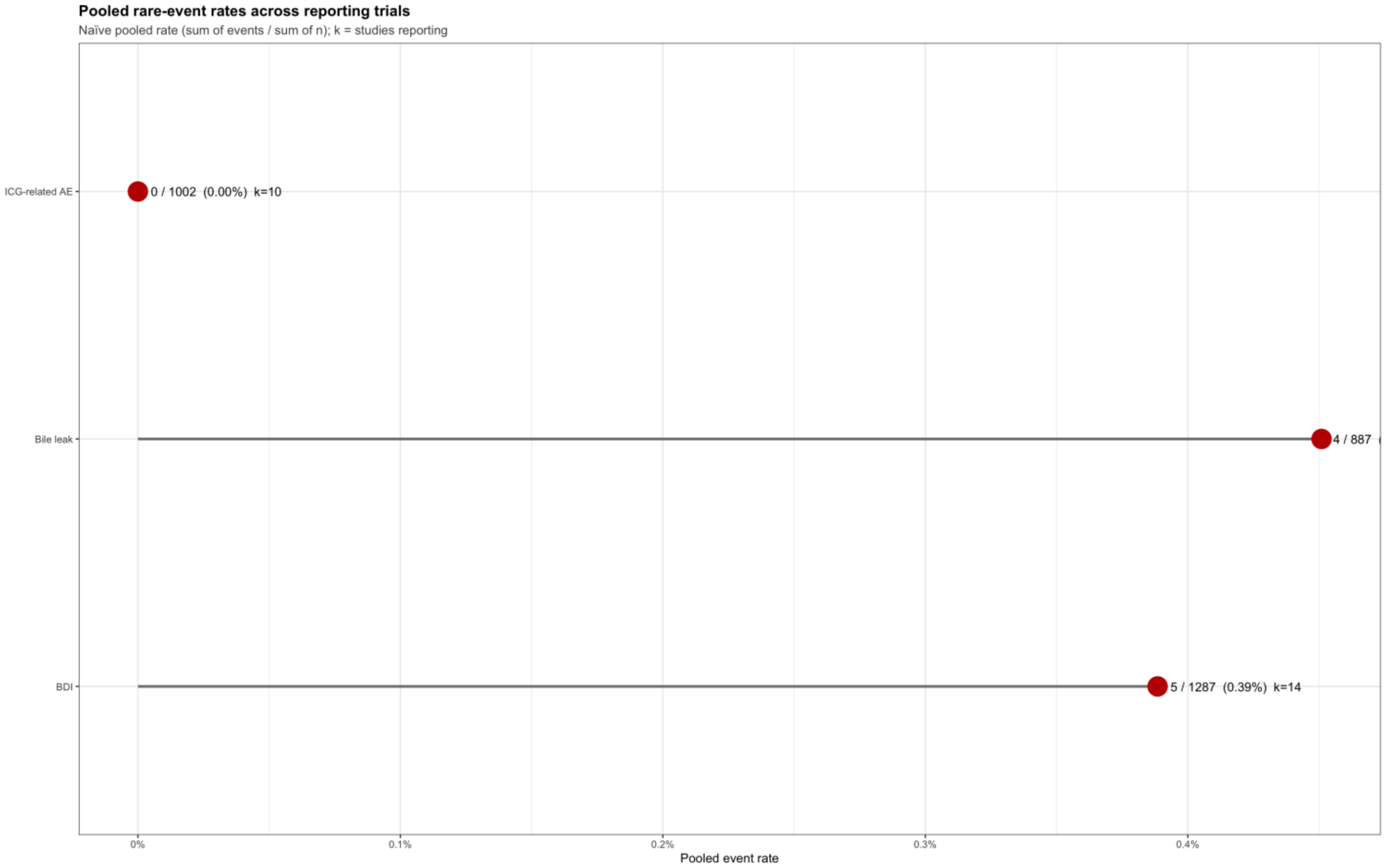

Supplementary Figure S3. Crude pooled rare-event rates across reporting trials. Naïve pooled event rates calculated as total events divided by total participants across studies reporting each endpoint. These values are descriptive and should not be interpreted as random-effects pooled estimates. BDI = bile duct injury; AE = adverse event.

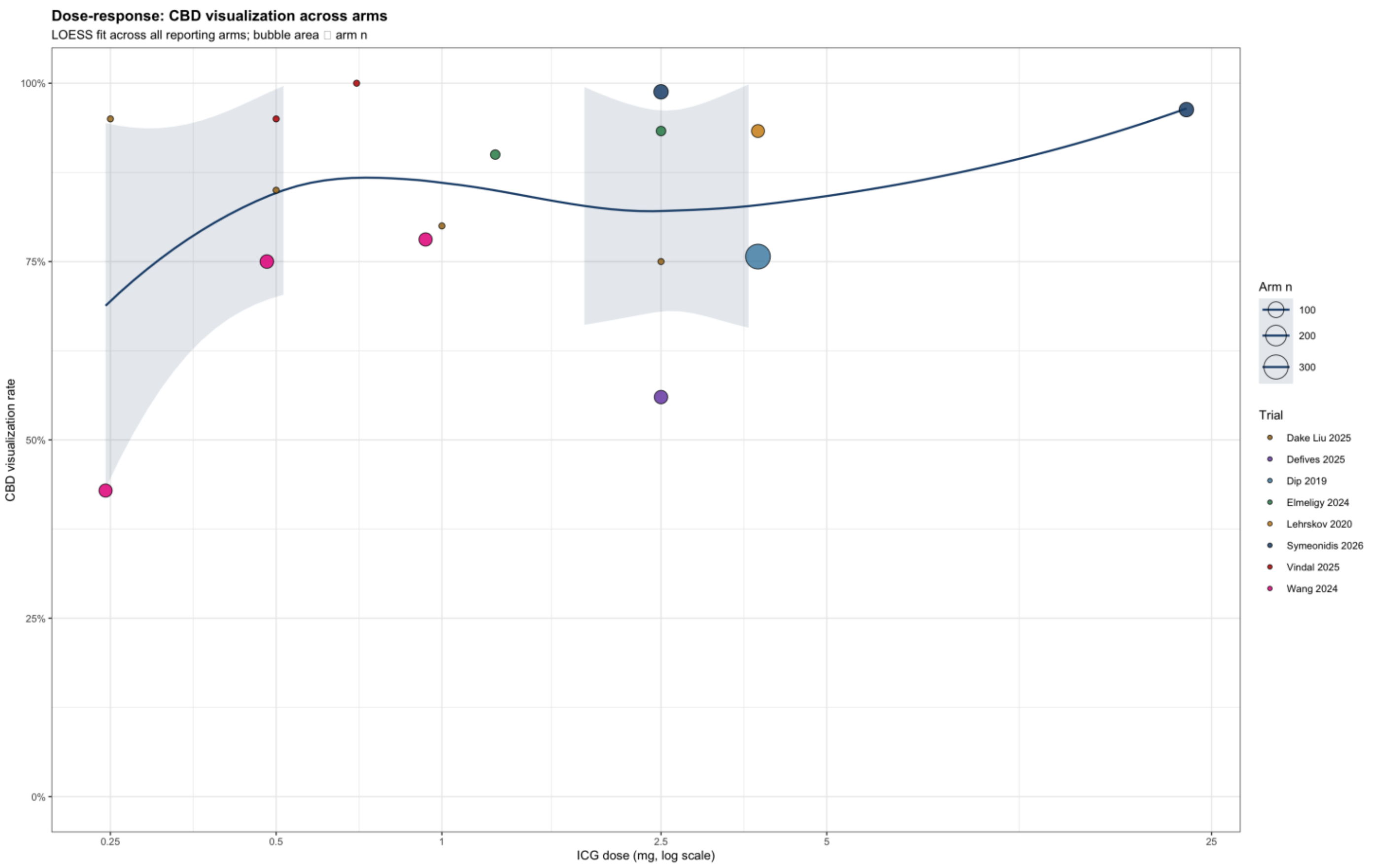

Supplementary Figure S4. Dose-response: CBD visualization across arms. Arm-level bubble plot of CBD visualization rate by ICG dose across reporting studies. Each bubble represents one study arm; bubble size reflects arm sample size. The LOESS curve provides descriptive visualization only and should not be interpreted as a formal meta-regression.

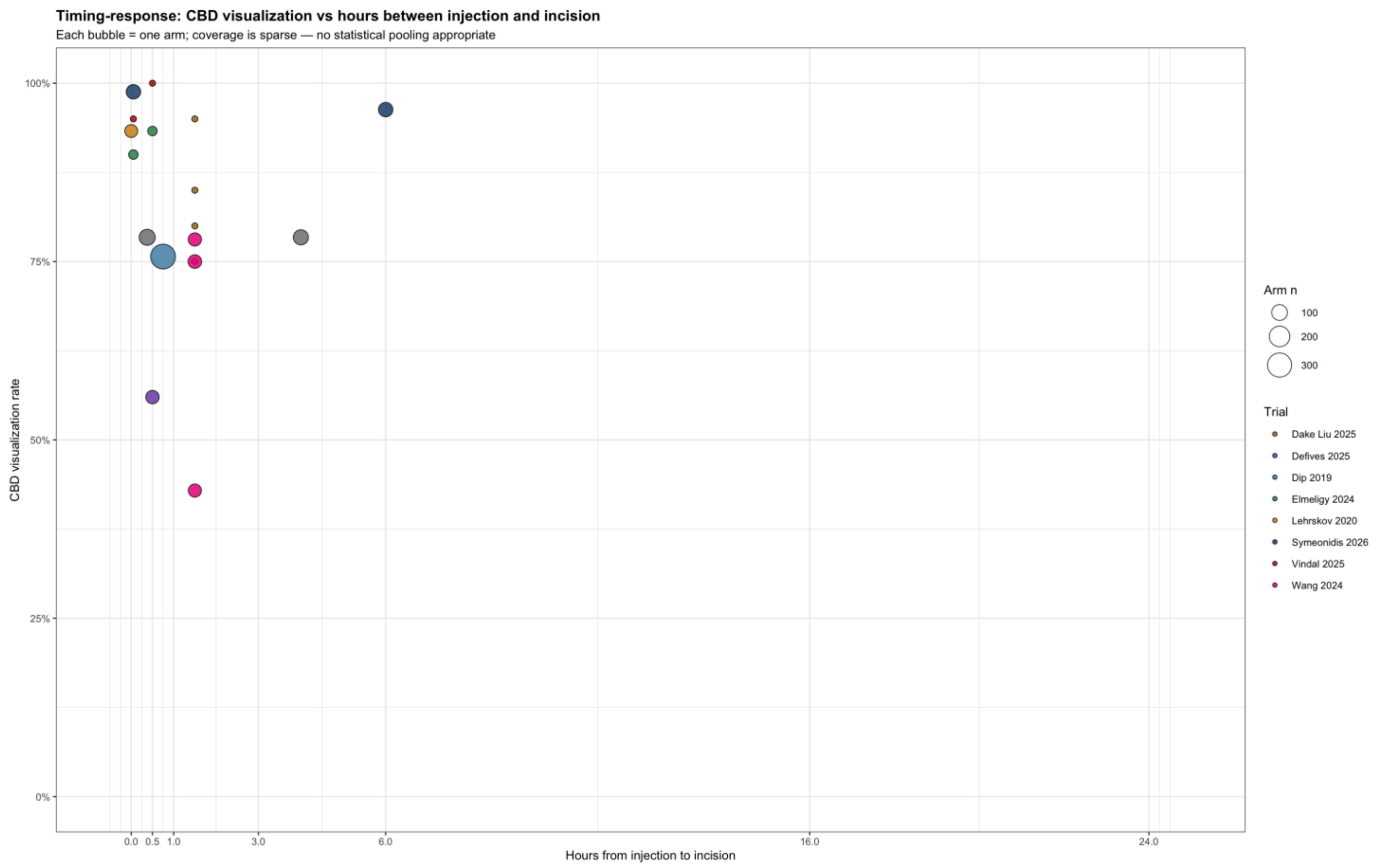

Supplementary Figure S5. Timing-response: CBD visualization versus injection-to-incision interval. Arm-level bubble plot of CBD visualization rate by time from ICG injection to incision. Bubble size reflects arm sample size. Data are clustered in short preoperative windows, limiting inference regarding delayed timing strategies.

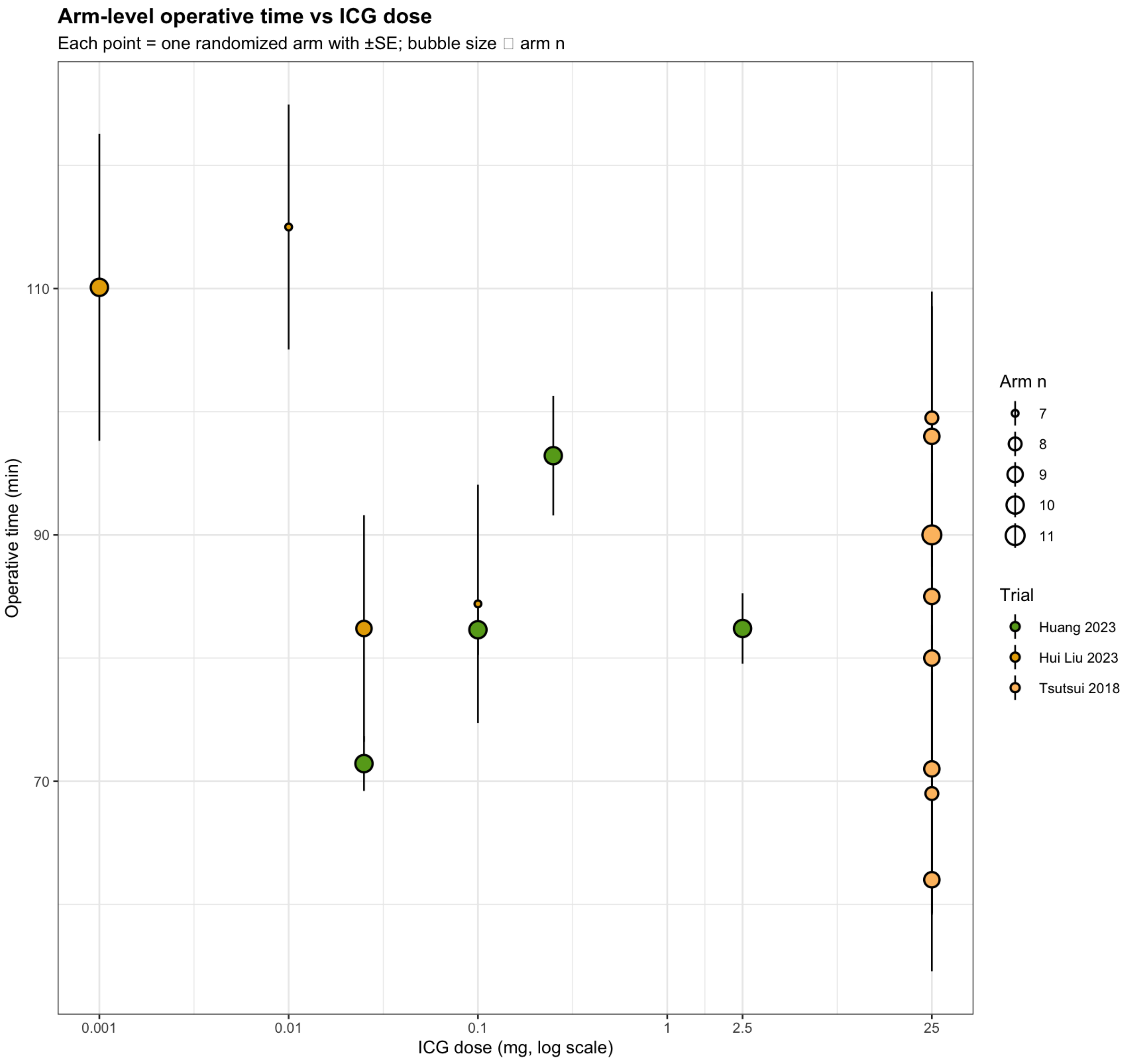

Supplementary Figure S6. Arm-level operative time versus ICG dose. Arm-level operative-time plot across dose/timing studies. Each point represents one randomized ICG arm with standard error bars where available; bubble size reflects arm sample size. This figure is descriptive and does not support a clear dose-dependent operative-time effect.

GRADE certainty of evidence: ICG fluorescence cholangiography in laparoscopic cholecystectomy

Randomized evidence started at high certainty; each domain shows the levels downgraded

Comparison	Outcome	Risk of bias	Inconsistency	Indirectness	Imprecision	Publication bias	Certainty
ICG fluorescence vs white-light imaging	Pre-dissection CBD visualization	−1	NA	0	0	ND	⊕⊕⊕◯ Moderate
	Pre-dissection CHD visualization	−1	NA	0	0	ND	⊕⊕⊕◯ Moderate
	Pre-dissection cystic duct visualization	−1	NA	0	0	ND	⊕⊕⊕◯ Moderate
	Post-dissection CBD visualization	−1	NA	0	0	ND	⊕⊕⊕◯ Moderate
	Post-dissection CHD visualization	−1	NA	0	0	ND	⊕⊕⊕◯ Moderate
	Post-dissection cystic duct visualization	−1	NA	0	−1	ND	⊕⊕◯◯ Low
	Time to critical view of safety	−1	−1	0	−1	ND	⊕◯◯◯ Very low
	Total operative duration	−1	0	0	−1	ND	⊕⊕◯◯ Low
	Conversion to open surgery	−1	0	0	−2	ND	⊕◯◯◯ Very low
	Bile duct injury	−1	0	0	−2	ND	⊕◯◯◯ Very low
	Postoperative morbidity	−1	−1	0	−1	ND	⊕◯◯◯ Very low
	Length of hospital stay	−1	0	0	−1	ND	⊕⊕◯◯ Low
IV ICG vs radiographic IOC: elective surgery	Critical-junction visualization	−1	0	0	−1	ND	⊕⊕◯◯ Low
	Cholangiography procedure duration	−1	0	0	0	ND	⊕⊕⊕◯ Moderate
	Total operative duration	−1	0	0	−1	ND	⊕⊕◯◯ Low
	Bile duct injury	−1	0	0	−2	ND	⊕◯◯◯ Very low
IV ICG strategy vs conventional IOC strategy: acute cholecystitis	Time to critical view of safety	−2	NA	0	−1	ND	⊕◯◯◯ Very low
	Biliary anatomical visualization	−2	NA	0	−1	ND	⊕◯◯◯ Very low
Intracholecystic vs intravenous ICG	Duct-specific anatomical visualization	−2	−1	0	−1	ND	⊕◯◯◯ Very low
	Hepatic background fluorescence	−1	0	0	−1	ND	⊕⊕◯◯ Low
	Total operative duration	−1	0	0	−1	ND	⊕⊕◯◯ Low
	Route-related adverse events & biliary complications	−1	0	0	−2	ND	⊕◯◯◯ Very low
Dose and timing comparisons	0.05 vs 2.5 mg at induction: CBD-to-liver fluorescence ratio	−2	NA	0	−1	ND	⊕◯◯◯ Very low
	0.1 mg vs other doses (<=30 min preop): CBD-to-liver contrast	−1	NA	0	−1	ND	⊕⊕◯◯ Low
	10-25 µg vs other microdoses: CBD-to-liver contrast	−1	NA	0	−1	ND	⊕⊕◯◯ Low
	0.25 mg vs higher doses: subjective image quality	−1	NA	0	−1	ND	⊕⊕◯◯ Low
	BMI-adjusted strategies: duct-identification rates	−1	NA	0	−1	ND	⊕⊕◯◯ Low
	~3 h vs 15-30 min preop: hepatic background interference	−1	NA	0	−1	ND	⊕⊕◯◯ Low
	15 h vs other intervals (25 mg): contrast and visibility	−1	NA	0	−1	ND	⊕⊕◯◯ Low
	0.05 mg at induction vs 0.25 mg/kg 16 h preop: CBD-to-liver ratio	−1	NA	0	−1	ND	⊕⊕◯◯ Low

Downgrade No downgrade · Serious (−1) · Very serious (−2) · Not assessable

Supplementary Figure S7. GRADE assessment of certainty of evidence for ICG fluorescence cholangiography during laparoscopic cholecystectomy. Abbreviations: GRADE, Grading of Recommendations Assessment, Development and Evaluation; ICG, indocyanine green; IOC, intraoperative cholangiography; BMI, body mass index; NA, not assessable between studies; ND, not downgraded. Assessment conventions: Randomized evidence started at high certainty. Domain entries indicate no downgrade (0), a one-level downgrade for serious concerns (−1), or a two-level downgrade for very serious concerns (−2).
